# CAR T cells targeting tumor-associated exons of glypican 2 regress neuroblastoma in mice

**DOI:** 10.1016/j.xcrm.2021.100297

**Published:** 2021-06-01

**Authors:** Nan Li, Madeline B. Torres, Madeline R. Spetz, Ruixue Wang, Luyi Peng, Meijie Tian, Christopher M. Dower, Rosa Nguyen, Ming Sun, Chin-Hsien Tai, Natalia de Val, Raul Cachau, Xiaolin Wu, Stephen M. Hewitt, Rosandra N. Kaplan, Javed Khan, Brad St Croix, Carol J. Thiele, Mitchell Ho

**Affiliations:** 1 Laboratory of Molecular Biology, Center for Cancer Research, National Cancer Institute, National Institutes of Health, Bethesda, MD 20892, USA; 2 Genetics Branch, Center for Cancer Research, National Cancer Institute, National Institutes of Health, Bethesda, MD 20892, USA; 3 Mouse Cancer Genetics Program, Center for Cancer Research, National Cancer Institute, Frederick, MD 21702, USA; 4 Pediatric Oncology Branch, Center for Cancer Research, National Cancer Institute, National Institutes of Health, Bethesda, MD 20892, USA; 5 Center for Molecular Microscopy, Center for Cancer Research, National Cancer Institute, National Institutes of Health, Frederick, MD 21702, USA; 6 Cancer Research Technology Program, Leidos Biomedical Research, Inc., Frederick, MD 21702, USA; 7 Data Science and Information Technology Program, Leidos Biomedical Research, Frederick, MD 21702, USA; 8 Laboratory of Pathology, Center for Cancer Research, National Cancer Institute, National Institutes of Health, Bethesda, MD 20892, USA

**Keywords:** glypican 2, pediatric cancers, immunotherapy, tumor-specific T cells, antibodies, electron microscopy, heparan sulfate proteoglycans, chimeric antigen receptor (CAR) T cells, neuroblastoma, epitopes

## Abstract

Targeting solid tumors must overcome several major obstacles, in particular, the identification of elusive tumor-specific antigens. Here, we devise a strategy to help identify tumor-specific epitopes. Glypican 2 (GPC2) is overexpressed in neuroblastoma. Using RNA sequencing (RNA-seq) analysis, we show that exon 3 and exons 7–10 of GPC2 are expressed in cancer but are minimally expressed in normal tissues. Accordingly, we discover a monoclonal antibody (CT3) that binds exons 3 and 10 and visualize the complex structure of CT3 and GPC2 by electron microscopy. The potential of this approach is exemplified by designing CT3-derived chimeric antigen receptor (CAR) T cells that regress neuroblastoma in mice. Genomic sequencing of T cells recovered from mice reveals the CAR integration sites that may contribute to CAR T cell proliferation and persistence. These studies demonstrate how RNA-seq data can be exploited to help identify tumor-associated exons that can be targeted by CAR T cell therapies.

## Highlights

RNA-seq analysis identifies tumor-associated exons of glypican 2 (GPC2)
The CT3 antibody specific for tumor-associated exons has been isolated
The complex structure of CT3 with GPC2 is visualized by electron microscopy
CT3-derived CAR T cells regress neuroblastoma in mice

## Graphical abstract

**Figure:**
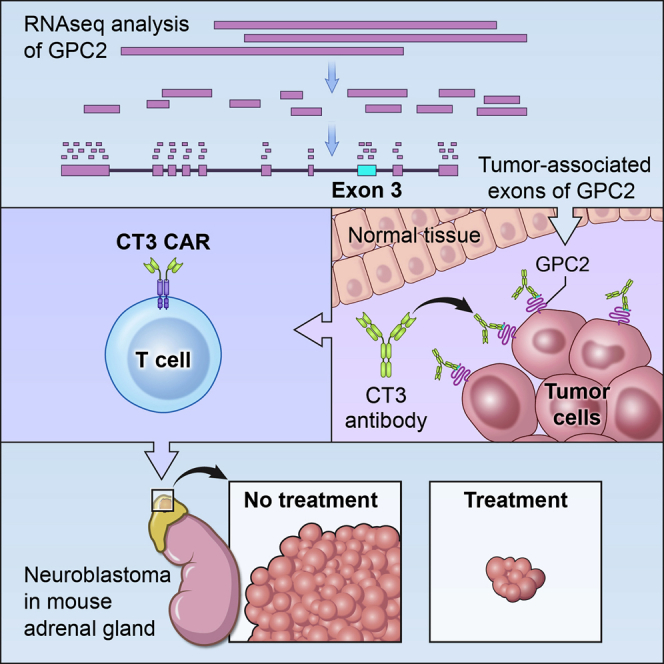

## Teaser

Li et al. exploit RNA-seq data to identify tumor-associated exons of glypican 2 (GPC2) that can be targeted by a monoclonal antibody (CT3). The complex structure of CT3 with GPC2 is visualized by electron microscopy. CT3-derived CAR T cells regress neuroblastoma in mice.

## Introduction

Chimeric antigen receptors (CARs) genetically engineered into T cells have become a new class of therapeutics for treating cancer. A CAR molecule contains an antigen-binding domain, in addition to T cell signaling and co-stimulatory domains. CAR T cell therapy has achieved impressive efficacy in treating B cell leukemia and lymphoma, as evidenced by the approval of Kymriah and Yescarta.^1^, ^2^, ^3^, ^4^ The success is largely due to the choice of the target, CD19.^5^ Ideally, CAR targets are antigens uniformly expressed on tumor cells but not normal cells, are shared by many cancer patients, and contribute to cancer oncogenic signaling, such that downregulation would inhibit cancer growth. Although CD19 is expressed in both malignant B cells and healthy B cells, B cell aplasia caused by CAR T cell treatment can be managed. However, solid tumors arise from organs or tissues that are indispensable, making them one of the greatest barriers for treating solid tumors.

Tumor antigens can be classified as either tumor specific (found on tumor cells with no or little expression on normal cells) or tumor associated (overexpressed on tumor cells with low-level expression on normal cells).^5^ Tumor-specific antigens can derive from tumor-specific mutations or include oncofetal proteins that are expressed during early development and silenced in adult normal tissues but aberrantly expressed in cancer cells, and testis-associated antigens. Glypicans (GPCs) are a family of heparan sulfate (HS) proteoglycans that are bound to the cell surface by a glycosylphosphatidylinositol (GPI) anchor.^6^^,^^7^ Certain GPCs, such as GPC3 and GPC2, are oncofetal antigens that are expressed in early development and largely silenced in adult normal tissues but upregulated in epithelial solid tumors.^7^, ^8^, ^9^, ^10^ We and others have demonstrated that GPC2 is highly expressed in neuroblastoma (NB), one of the deadliest childhood cancers, and minimally expressed in normal tissues, making it an attractive candidate for CAR T cell therapy.^11^, ^12^, ^13^ Despite intensified cytotoxic therapy and immunotherapy, outcomes for patients with high-risk NB remain poor, with a long-term survival rate of <50%.^14^^,^^15^ GPC2 is implied in the regulation of *MYCN*,^12^ which has long been associated with high-risk disease and poor outcome in NB.^12^^,^^16^ Interestingly, tumor and normal tissues express different GPC2 transcripts,^13^ indicating the possible existence of highly tumor-selective GPC2 sequences that may be useful for GPC2-targeted immunotherapies.

In this study, we devised a strategy to identify tumor-specific epitopes. We used an exon-based RNA sequencing (RNA-seq) analysis to identify “tumor-associated exons” of GPC2. We then identified a monoclonal antibody (mAb), which we called CT3, that recognizes tumor-associated exons 3 and 10 of GPC2. Furthermore, we developed CAR T cells based on CT3 that showed potent activity against preclinical models of metastatic and localized established NB in mice.

## Results

### Isolation of an anti-GPC2 antibody with undetectable binding to normal vital organs

**Figure 1:**
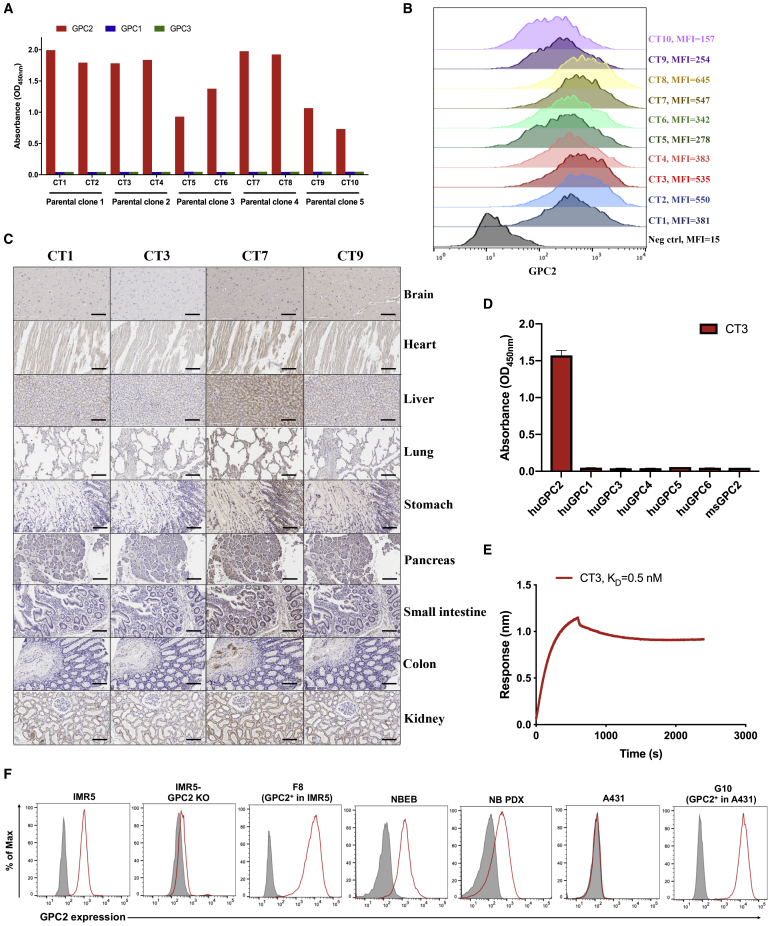
Isolation of GPC2-specific mouse mAbs using hybridoma technology (A) Ten mouse mAbs (CT1–CT10) from 5 parental clones only bind to human glypican 2 (GPC2), but not to human GPC1 and GPC3 by ELISA. (B) Flow cytometry comparing 10 mouse mAbs at 10 μg/mL shows increased binding of each to GPC2^+^ LAN5 neuroblastoma (NB) cells compared to negative control immunoglobulin G (IgG). (C) IHC staining with 1 μg/mL of mAb reveals that CT3 has lowest reactivity with human vital organs compared with CT1, CT3, and CT9 mAbs. Scale bar, 200 μm. (D) CT3 shows binding to human GPC2, but not to other human GPCs and mouse GPC2. 1 μg/mL of CT3 was used for ELISA. (E) Octet kinetic analysis for the interaction between CT3 and human GPC2. The K_D_ value was 0.5 nM. (F) Cell surface GPC2 expression in NB cells, including IMR5 and NBEB, GPC2-overexpressing cells including G10 and F8, NB patient-derived xenograft (PDX) cells, and GPC2^−^ cells A431 and GPC2 knockout (KO)-IMR5 cells. Shaded gray peaks represent the cell surface staining with isotype control, and peaks colored in red represent the cell surface staining of GPC2 using CT3 at 10 μg/mL.

Although glypican members share ∼25% amino acid similarity, their C-terminal regions close to the cell membrane are low in sequence similarity.^17^ It has been indicated that CARs targeting membrane-proximal epitopes may have better antitumor activity than those incorporating membrane-distal binding domains.^18^^,^^19^ Thus, we immunized mice using the C-terminal region (exon 10) of GPC2 and isolated mAbs with membrane-proximal GPC2-specific epitopes. As shown in Figure 1A, 10 mAbs (CT1–CT10) were recovered from 5 parental hybridoma clones and all specifically bound to human GPC2. All of the mAbs were then assessed for their binding affinity to cell surface GPC2 using the LAN5 NB cell line (Figure 1B). The mAbs from parental clones 1, 2, and 4 showed strong binding to GPC2. Next, we determined the reactivity of the four representative mAbs to a panel of human healthy tissues by immunohistochemistry (IHC). Notably, no detectable GPC2 staining with CT3 derived from parental clone 2 was observed in vital organs such as the brain, heart, liver, and lung, whereas binding was detected in multiple tissues stained with the other mAbs, including CT1 and CT7 derived from parental clones 1 and 4, respectively (Figure 1C). In addition, the absence of CT3 binding to GPC2 in other tissues (except testis) was demonstrated by immunostaining a comprehensive panel of normal tissues (Figure S1; Table S1).

Furthermore, we found that CT3 was unable to recognize other human GPC family members (Figure 1D). An Octet kinetic analysis revealed that CT3 binds to GPC2 with high affinity (the equilibrium dissociation constant [K_D_] value of 0.5 nM) (Figure 1E). We also examined the binding of CT3 to GPC2 in live cells using flow cytometry. CT3 was able to surface stain GPC2-expressing NB cells (IMR5 and NBEB), NB patient-derived xenograft (PDX) cells, GPC2-overexpressing IMR5 cells (F8), and GPC2-overexpressing A431 cells (G10) (Figure 1F). Conversely, CT3 did not stain GPC2 knockout (KO) IMR5 cells or GPC2^−^ A431 cells, suggesting that this binding is antigen specific. To summarize, we successfully identified a mAb, CT3, that selectively binds to GPC2 in human cancer cells with undetectable reactivity in almost all human normal tissues.

### GPC2 exon 3 has the least expression in normal human tissues

**Figure 2:**
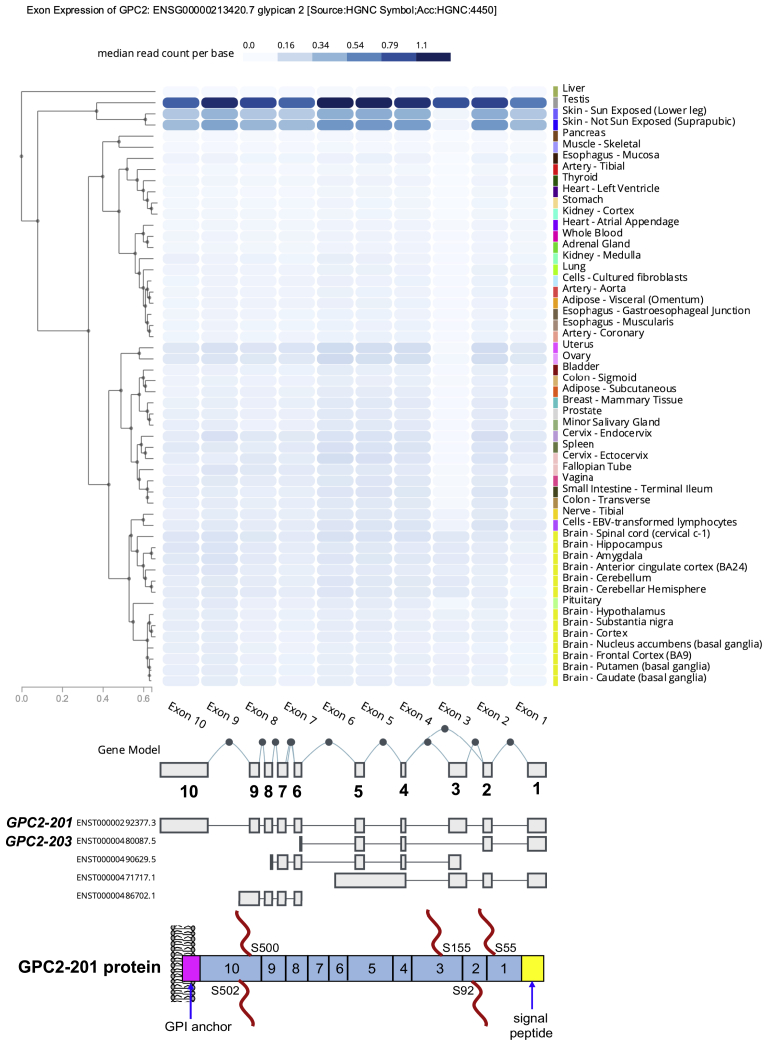
GPC2 exon expression profile in the GTEx database GTEx data analysis report as the median read counts per base (i.e., the median raw read counts normalized by exon length for each exon).

An improved therapeutic window in cancer treatment can be achieved by specifically targeting a sequence such as an isoform that is enriched in tumors.^20^ To investigate this possibility for GPC2, we examined the GPC2 mRNA transcripts in the Ensembl database. So far, 5 GPC2 transcripts have been identified, but only 2 of them, *GPC2-201* and *GPC2-203*, contain open reading frames (ORFs) (Figure 2; Table S2). *GPC2-201*, encoding the longest form of GPC2 protein that contains exons 1–10 with 5 hypothetic HS chains, is the isoform overexpressed in pediatric cancers.^12^^,^^13^ *GPC2-203*, which has a smaller ORF containing only exons 1–2 and 4–6, was barely detected in NB,^13^ indicating that exon 3 and exons 7–10 encode the fragments that are highly enriched in tumors. Next, we analyzed GPC2 exon expression using data from the Genotype-Tissue Expression (GTEx) project, which include 17,382 RNA-seq samples from 948 donors across 54 non-disease tissues.^21^ Interestingly, we found that exon 3 was found only in the testis and modestly expressed in the brain, with extremely low levels of expression in all other normal tissues (Figure 2). Most important, exon 3 showed the lowest expression in all normal human tissues compared with all other exons. Overall, exon 3 of *GPC2-201* showed the lowest expression in normal tissues, indicating that this region of GPC2 could be targeted to enhance the safety and specificity of immune-based therapies for treating solid tumors.

### CT3 binds to tumor-associated epitopes on GPC2

**Figure 3:**
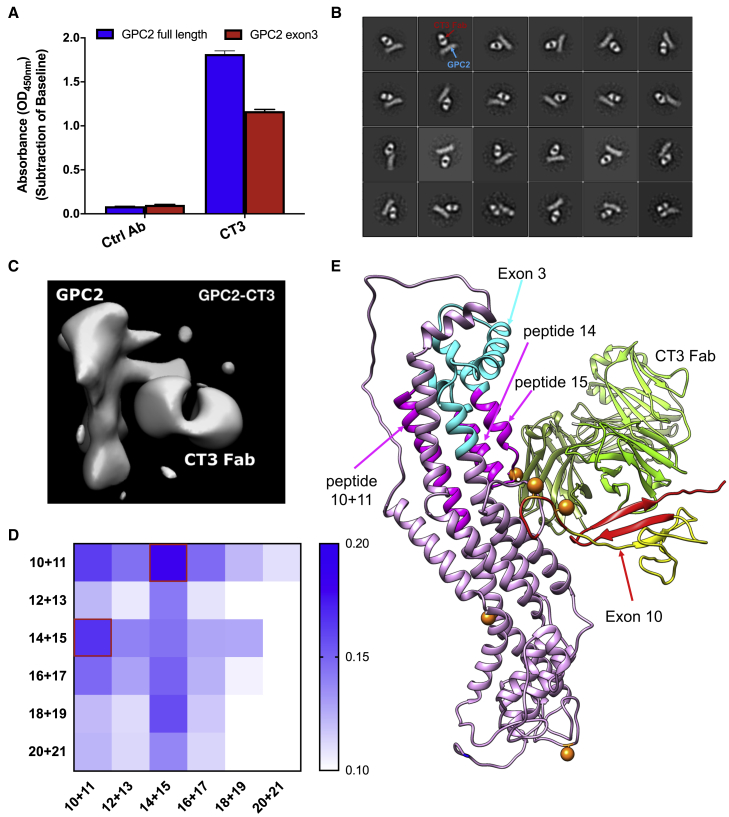
Characterization and binding epitope of the CT3 mAb (A) CT3 binds not only to the full-length GPC2 protein, but also to the exon 3 of GPC2; 1 μg/mL CT3 was used for ELISA. (B) Representative 2D class averages of GPC2-CT3 Fab complex. (C) An enlarged view of a 2D class average of GPC2 in complex with CT3 Fab. (D) Epitope mapping of CT3 in exon 3 of GPC2 using a 2 × 2 matrix study. 5 μg/mL peptide mixtures were coated in the assay plate and 1 μg/mL CT3 was used in the ELISA experiment. (E) A ribbon diagram of CT3 Fab and GPC2 with highlighted peptide regions that may contain the CT3′s binding epitope. Peptides 10, 11, 14, and 15 are colored magenta.

To determine whether any of our antibodies may react with tumor-associated regions of GPC2, we performed ELISA using different GPC2 fragments derived from exons 3 and 10. Surprisingly, CT3 strongly bound not only to the C-terminal region (exon 10) that was used for immunization (Figure S2A) but also to the recombinant exon 3 fragment of GPC2 (Figure 3A), which helps to explain the reduced reactivity of CT3 in normal tissues. To validate the binding epitope of CT3 on GPC2, we conducted negative stain electron microscopy (EM) to visualize the structure of the GPC2:CT3 antibody-binding fragment (Fab) complex. The representative EM images in Figure 3B show that CT3 formed a stable and rigid complex with GPC2. To obtain maps with a higher local resolution, we performed particle subtraction, followed by additional classifications and three-dimensional (3D) refinement. The final 3D reconstruction, with a resolution of 21 Å (Figure 3C), showed that CT3 interacts with epitopes from both exons 10 and 3 of GPC2 (Figure S2B). In particular, the exon 3 peptide is spatially adjacent to the exon 10 peptide based on the 3D structure, and both regions may be close to the cell membrane.

To identify the CT3′s epitope, we made a GPC2 peptide library that comprises 18 amino acid peptides with 9 amino acid overlap and performed epitope mapping using the 12 peptides (peptides 10–21) for exon 3. The sequences are listed in Table S3. First, we determined CT3′s binding to each peptide by ELISA; however, no obvious reactivity to any linear peptide was found (Figure S2C), indicating that CT3 may recognize a conformational epitope in exon 3. Second, we performed a 2 × 2 matrix study by combining the 12 peptides. As shown in Figure 3D, CT3 showed an appreciably stronger binding to the mixture of peptides 10–11 and peptides 14–15 than other combinations. We predicted that the residues 148–162 (LRDFYGESGEGLDDT), which are found in peptides 14 and 15, could contain the CT3′s epitope in exon 3 of GPC2 based on the EM structure. Interestingly, the helix that comprises peptides 10–11 is close to peptide 14 (Figure 3E), suggesting that the binding of CT3 to exon 3 needs the interaction between these 2 regions. Overall, we demonstrate that CT3 binds, at least in part, to a region of GPC2 encoded by exon 3, a unique sequence predominantly expressed in tumors but undetectable in most healthy tissues.

### GPC2 is expressed in multiple pediatric cancers

**Figure 4:**
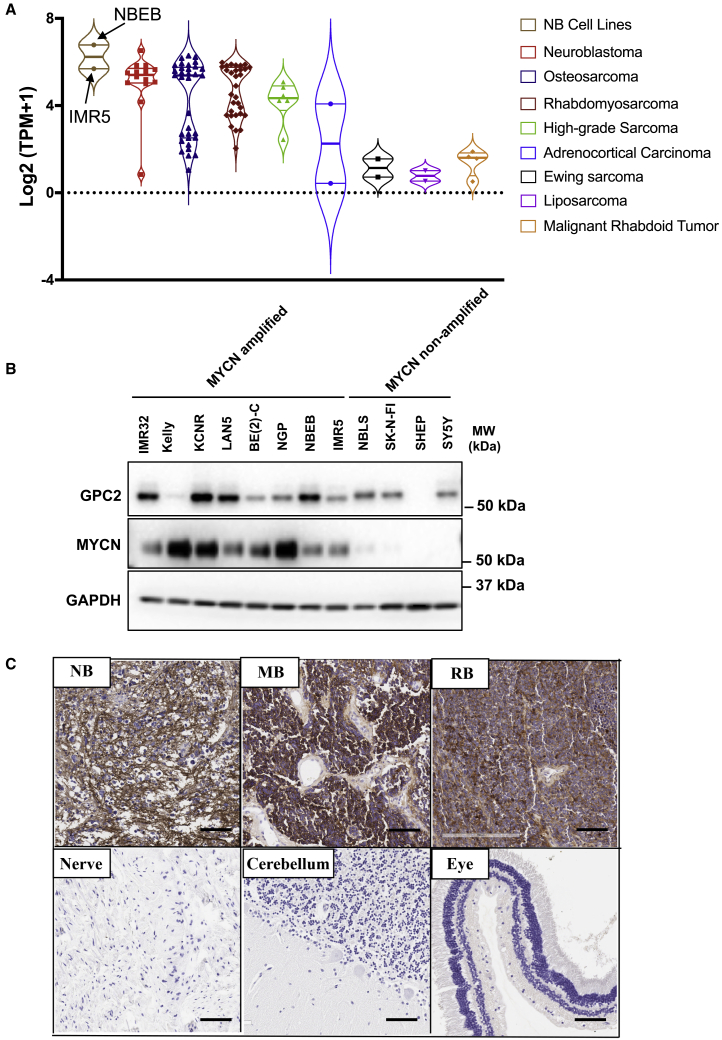
GPC2 expression in pediatric cancers (A) *GPC2* mRNA expression in RNA-seq dataset of orthotopic PDXs of pediatric solid tumors. *GPC2* mRNA levels in NB cell lines NBEB and IMR5 are included. (B) Western blotting with CT3 was performed to detect GPC2 protein expression in 8 *MYCN*-amplified NB cell lines (IMR32, Kelly, KCNR, LAN5, BE(2)C, NGP, NBEB, IMR5), and 4 *MYCN* non-amplified NB cell lines (NBLS, SK-N-FI, SK-N-MM, SHEP, SY5Y). (C) IHC data show increased GPC2 expression in NB, medulloblastoma (MB), and retinoblastoma (RB) compared with normal peripheral nerve, cerebellum, and eye tissues. 1 μg/mL CT3 was used for IHC. Scale bar, 200 μm.

Consistent with our prior findings showing that a majority of NBs express GPC2,^12^ *GPC2* mRNA expression was detected in NB cell lines, with the highest level in NBEB cells and the lowest level in SKNAS cells (Figure S3A). We used the published RNA-seq dataset of orthotopic PDXs of pediatric solid tumors^22^ to examine the *GPC2* mRNA levels. The data revealed appreciable *GPC2* mRNA expression in osteosarcoma, rhabdomyosarcoma, and high-grade sarcoma in addition to NB (Figure 4A). In addition, the NBEB and IMR5 cell line models used in the present study for mouse testing show similar *GPC2* mRNA expression as the PDXs. CT3 was then used to analyze GPC2 levels in *MYCN* non-amplified and *MYCN*-amplified NB cell lines. As shown in Figures 4B and S3B, GPC2 protein was observed in nearly 90% (7/8) of MYCN-amplified NB cell lines; it was only detected in 33% (3/9) of *MYCN* non-amplified NB cell lines. By comparison, GPC2 protein expression was not found in the non-NB cell lines 293T, HeLa, and ARPE19. The specificity of the commercially available antibody against the N-Myc protein was validated by western blot in Figure S3C. Lastly, GPC2 expression was noticeably higher in the NB PDX than IMR5 cells cultured as monolayers *in vitro* (Figure S3D).^23^

Next, we used CT3 to validate GPC2 protein expression in pediatric cancers. GPC2 was found to be expressed in 95% (19/20) of NB tissues (Figures 4C and S4A). Moreover, GPC2 levels were elevated in 78% of medulloblastoma (MB) tissues (7/9) compared with normal brain samples (Figures 4C and S4B). We also found strong GPC2 staining in 78% of retinoblastoma (RB) tissues (7/9), but none in the non-cancerous sclera or retina samples (Figures 4C and S4C). Overall, these data show that GPC2 is highly expressed in MB and RB, indicating GPC2 as a potential target in these aggressive pediatric embryonal cancers.

### CT3 CAR T cells specifically kill GPC2^+^ tumor cells

**Figure 5:**
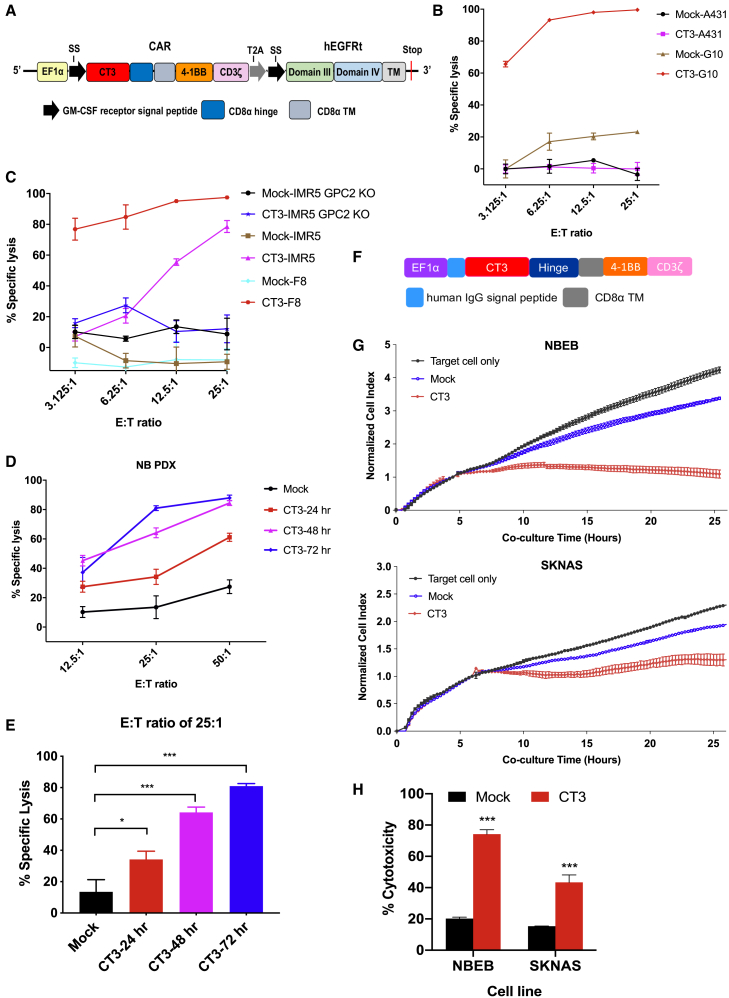
Cytolytic activity of CT3 CAR T cells *in vitro* (A) schematic of a CAR construct based on the CT3 scFv sequence. (B) CT3 CAR T cells potently lyse GPC2-overexpressing A431 cells (called G10) without affecting GPC2^−^ negative A431 cells after 24 h of co-culture. (C) Cytolytic activity of CT3 CAR T cells in IMR5 cells, GPC2-overexpressing IMR5 cells (called F8), as well as GPC2 knockout-IMR5 cells after 24 h of co-culture. (D) CT3 CAR T cells efficiently lyse NB PDX #3 cells in a time-dependent manner. CAR T cells and PDX cells were co-cultured for 24, 48, and 72 h. (E) The percentage of lysed PDX #3 cells after being co-cultured with CT3 CAR T cells for different time periods at an E:T ratio of 25:1. The cytolytic activity of CAR T cells from (B) to (E) was measured using a luciferase-based killing assay. (F) Schematic of the CT3 CAR construct that is used for xCELLigence real-time cell analysis. The construct was cloned into the pELPS-EF1α vector. The CD8α hinge was incorporated into the CAR. (G) NBEB and SKNAS NB cells were plated in E-plate and continuously monitored for growth using the xCELLigence for 5 h. The mock or CT3 CAR T cells were then added at E:T ratio of 1:1, and co-cultured for an additional 20 h. The cell index measurement was normalized to the time point of T cell addition. (H) The quantitation of cytotoxicity shown in (G) at the end of the assay. The target cell alone was used as a control for normalization. Values represent means ± SEMs. ∗p < 0.05; ∗∗∗p < 0.001.

To evaluate the therapeutic potential of the CT3 mAb, we generated a CAR using the single-chain variable fragment (scFv) derived from CT3 and included a 4-1BB endodomain (Figure 5A). In addition, a truncated human epidermal growth factor receptor (hEGFRt) containing the binding epitope of the anti-EGFR mAb cetuximab was added into the lentiviral construct to allow cell tracking and to function as an off “kill switch.”^24^ We assessed how the density of GPC2 expression in target cells affects the antitumor activity of CT3 CAR T cells. As shown in Figures 5B and 5C, CT3 CAR T cells killed nearly 70% of G10 and F8 cells, which express high levels of GPC2, even at the effector T cell to target cell (E:T) ratio of 3:1. IMR5 cells, which express lower levels of GPC2, showed comparable cytolytic activity of CT3 CAR T cells at higher E:T ratios. By contrast, minimal cell lysis was observed in GPC2^−^ A431 cells and GPC2 KO-IMR5 cells when co-cultured with CT3 CAR T cells, demonstrating target-dependent CAR T cell killing. Furthermore, the antitumor activity of CT3 CAR T cells was evaluated against cells derived from the NB PDX as they can be cultured *in vitro* and were engineered to express luciferase. We observed a gradual increase in the cytolytic activity of CT3 CAR T cells in a time-dependent manner (Figure 5D). At the E:T ratio of 25:1, the percentage of lysed target cells increased from 30% at 24 h to 80% at 72 h of co-culture (Figure 5E). Lastly, we used the xCELLigence technology to monitor CT3 CAR T cell-mediated killing in additional GPC2^+^ NB cell lines in real time. CT3 CAR T cells, but not mock T cells, substantially decreased the impedance of the target cell monolayer, which is indicative of cytolysis (Figures 5F and 5G). At the E:T ratio of 1:1, the percentage of cytotoxicity in NBEB cells and SKNAS cells was 74% and 43% at 25 h of co-culture (Figure 5H). This observation is consistent with the *GPC2* mRNA levels in the two cell lines. These results show that CT3 CAR T cells display highly selective cytotoxicity against GPC2^+^ NB cells, including PDX-derived tumor cells.

### CT3 CAR T cells exhibit a low level of tonic signaling

Antigen-independent tonic signaling by CARs can increase the differentiation and exhaustion of T cells, limiting their potency. Incorporation of 4-1BB endodomain in a lentiviral vector could reduce this functional exhaustion.^25^^,^^26^ Here, we examined tonic signaling from CT3 CAR T cells during *ex vivo* expansion. As diasialoganglioside GD2-targeting immunotherapies are under clinical and preclinical investigation in NB,^25^^,^^27^, ^28^, ^29^ we constructed a GD2-targeted 4-1BB CAR to serve as a positive control for *in vitro* comparison. T cell activation levels of GD2 and GPC2 (CT3) CAR T cells were similar to mock T cells on days 4 and 8 (Figures S5A and S5B). During days 8–11, GD2 and GPC2 (CT3) CAR T cells showed indistinguishable expression of exhaustion markers (PD-1, TIM-3, and LAG-3) compared with mock T cells (Figures S5C and S5D). Both CAR T cells showed lower rates of apoptosis than mock T cells (Figure S5E). In addition, GD2 and GPC2 (CT3) CAR T cells induced comparable levels of tumor cell killing and cytokine production following exposure to IMR5 cells (Figures S5F–S5H). Furthermore, we analyzed the T differentiation subsets consisting of stem cell-like memory T cells (T_SCM_: CD62L^+^CD45RA^+^CD95^+^), central memory T cells (T_CM_: CD62L^+^CD45RA^−^CD95^+^), effector memory T cells (T_EM_: CD62L^−^CD45RA^−^CD95^+^), and terminally differentiated effector memory T cells (T_EMRA_: CD62L^−^CD45RA^+^CD95^+^). We observed sustained frequencies of T_SCM_ in CD4^+^-GD2 and GPC2 (CT3) CAR T cells at days 4 and 11 after activation (Figure S5I) and increased percentages of T_SCM_ of both CAR T cells in the CD8^+^ T cell population during expansion (Figure S5J). T_SCM_ is known to be the least differentiated of the memory T cell subsets.^30^ By comparison, mock T cells are more differentiated as evidenced by the increased frequencies of T_EMRA_. These functional and phenotypic studies demonstrate that the current format of CT3 CAR exhibits a low level of tonic signaling.

### Persistent CT3 CAR T cells are selected and undergo clonal expansion

**Figure 6:**
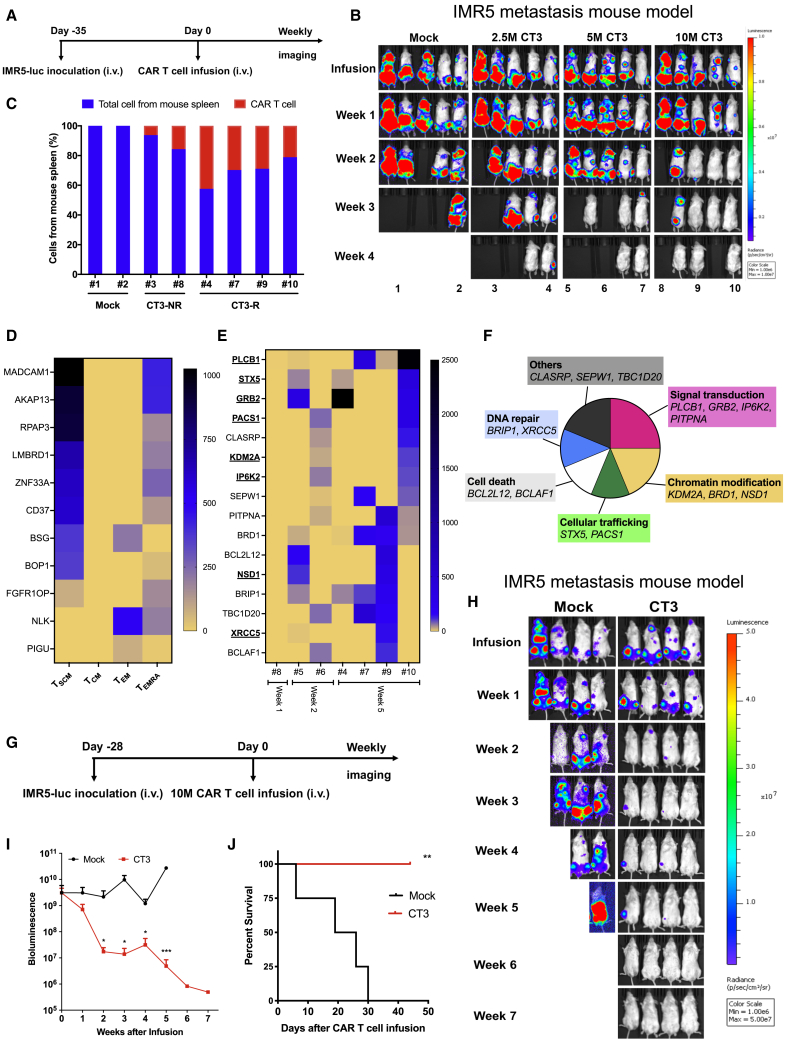
CT3 CAR T cells undergo *in vivo* selection eliminate tumor cells in the IMR5 xenograft mouse model (A) Experimental schematic of the IMR5 experimental metastasis xenograft mouse model. IMR5 tumor-bearing NSG mice were i.v. injected with 5 million (5M) mock T cells and different doses of CT3 CAR T cells including 2.5M, 5M, and 10M. (B) CT3 CAR T cells demonstrate potent antitumor activity and mediate complete regression of IMR5 xenograft tumors, especially at the 10M dose. (C) Detection of CAR vector-positive cells in the spleen of mice bearing tumors that were non-responsive (NR) or responsive (R) to CT3 CAR T cells. (D) eleven integrated genes are shared in at least 2 memory T cell subsets. Stem cell-like memory T cells (T_SCM_), central memory T cells (T_CM_), effector memory T cells (T_EM_), and terminally differentiated effector memory T cells (T_EMRA_). (E) The heatmap of enriched integrated genes from weeks 1 to 5 post-CT3 CAR T cell infusion. The genes found in the memory T cell subsets shown in Table S5 are bold and underlined. (F) The 16 genes shown in (E) are clustered based on their functions as shown in the pie chart. (G) Experimental schematic of the repeated metastatic IMR5 xenograft mouse model. IMR5 tumor-bearing NSG mice were i.v. injected with 10M mock T cells and CT3 CAR T cells. (H) Weekly image of mice treated with mock T cells or CT3 CAR T cells. (I) Tumor bioluminescence of mice in the mock and CT3 CAR groups shown in (H). (J) Kaplan-Meier survival curve showed 100% of mice in (H) survived post-CT3 CAR T cell infusion by week 7. Values represent means ± SEMs. ∗p < 0.05; ∗∗p < 0.01; ∗∗∗p < 0.001.

To evaluate the antitumor effects of CT3 CAR T cells *in vivo*, we generated an experimental metastatic NB model by inoculating luciferase-expressing IMR5 cells (IMR5-luc) into NOD/SCID/IL-2Rgc^null^ (NSG) mice intravenously (i.v.) via tail vein injection (Figure 6A). IMR5 xenograft tumors colonized tissues at clinically relevant sites such as the femur and spine (Figure S6A). Thirty-five days post-tumor cell inoculation, when the average bioluminescence intensity (BLI) of the tumors reached 5 × 10^9^, mice were randomized and infused with mock T cells and CT3 CAR T cells at various doses, including 2.5 million (2.5M), 5M, and 10M. As shown in Figures 6B and S6B, the groups that received CT3 CAR T cells showed evidence of decreased tumor burden. At the experimental endpoint, 20% of the mice in the 2.5M group and 40% of the mice in the 5M group experienced a complete tumor regression (i.e., tumor burden was undetectable by BLI). Furthermore, we noticed that the therapeutic effects of the CT3 CAR occurred faster in mice infused with the highest dose (10M) compared to the low-dose groups. Eleven days post-infusion, 80% of the mice in the 10M group appeared to have a decreased tumor burden, with tumors undetectable in 40% of the mice. We assessed the percentage of CAR T cells in the spleen using droplet digital PCR (ddPCR), a technique that allows measurement of the absolute copy number of CAR vector-positive cells. As shown in Figure 6C, 21.1%–42.4% of CAR vector-positive cells were detected in mice that responded to the CT3 CAR T cells (CT3-R), whereas 6.2%–15.7% of CAR vector-positive cells were found in mice that failed to respond (CT3-NR), demonstrating an inverse correlation between tumor burden and T cell persistence. Moreover, CT3 CAR T cells were detected and recovered from spleen and liver metastases of a treated mouse by flow cytometry (Figure S6C). The CT3 CAR T cells retrieved from mouse potently lysed IMR5 cells but not GPC2 KO-IMR5 cells (Figure S6D).

To analyze the molecular determinants of CT3 CAR T cell efficacy and persistence, we conducted genomic sequencing of the lentiviral integration sites of CAR T cells cultured *in vitro* as well as those recovered from the spleens and tumors of mice 1–5 weeks post-treatment. We have found thousands of integration sites from different memory T cell subsets (Figure S6E). Although no significantly enriched hotspot was found, 11 integrated genes were identified in at least 2 subsets, and most of these genes were shared between T_SCM_ and T_EMRA_ (Figure 6D). Consistently, the integration sites from pre-infusion cultured CT3 CAR T cells were randomly distributed (Figure S6F). Starting at week 2, however, CT3 CAR showed enrichment in clusters of genes that were largely overlapping in 2 mice (#9 and #10) that showed a fast and complete response even at week 2 post-infusion. Interestingly, 16 integration sites were enriched from weeks 1 to 5; these genes are important for signal transduction, chromatin modification, and so forth (Figures 6E and 6F; Table S5. While some of these genes (*GRB2*, *PACS1*, and *KDM2A*) are commonly found as integrated genes by lentiviral vectors,^31^ we found that some uncommon integration genes such as *PLCB1* and *BRD1* were enriched and shared in 3 of 4 mice at week 5 post-infusion.

We also analyzed the existence of CT3 CAR and its integration sites in various tissues from the same animal. For this study, the IMR5 tumor-bearing mice were infused with 5 million CT3 CAR T cells or control CAR T cells targeting GPC3, another GPC family member not expressed in NB (Figure S7A).^19^ As expected, CT3 CAR T cells suppressed tumor growth and control CAR T cells did not (Figure S7B). At weeks 2 and 3 post-infusion, CAR vector-positive cells were detected in nearly all of the collected tissues (liver, brain, spine, femur, and lymph nodes) from CT3 CAR T cell-treated mice, but were rarely found in corresponding tissues from the control CAR group (Figure S7C). Interestingly, the integrated sites of CT3 CAR were largely shared among different tissues of the same mouse, with the majority in spleen and liver, indicating the clonal expansion of CT3 CAR T cells in mice (Figure S7D; Table S6. By contrast, no integration into these genes was found in any tissues from mice treated with control CAR T cells. Furthermore, 22 of these 46 enriched or shared integrated genes (e.g., *PLCB1*) were detected in different memory T cell subsets with the preferences in T_SCM_ and T_EMRA_ cells (Figure S6G; Table S7. We found that persistent CT3 CAR T cells have integration sites related to specific genes shared in different mice at different time points. This indicates a potential selection pressure for integration sites in the genome for CAR T cell activation, survival, and clonal expansion *in vivo*.

### CT3 CAR T cells regress NB xenografts in mice

Next, we evaluated the persistence of CT3 CAR T cells using the metastatic IMR5 mouse model over a longer period. When the average BLI of the tumors reached 5 × 10^9^ post-IMR5-luc tumor inoculation (i.v.), the mice received either 10M mock T or 10M CT3 CAR T cells (Figure 6G). CT3 CAR T cells regressed tumors in 100% of mice, while continued tumor growth led to the death of all of the mice in the mock group by the end of the experiment (7 weeks after CAR T cell infusion) (Figures 6H and 6I). In this study, 100% overall survival was achieved in the CT3 CAR T cell group by week 6, at which time no survivors remained in the mock T cell group (Figure 6J).

**Figure 7:**
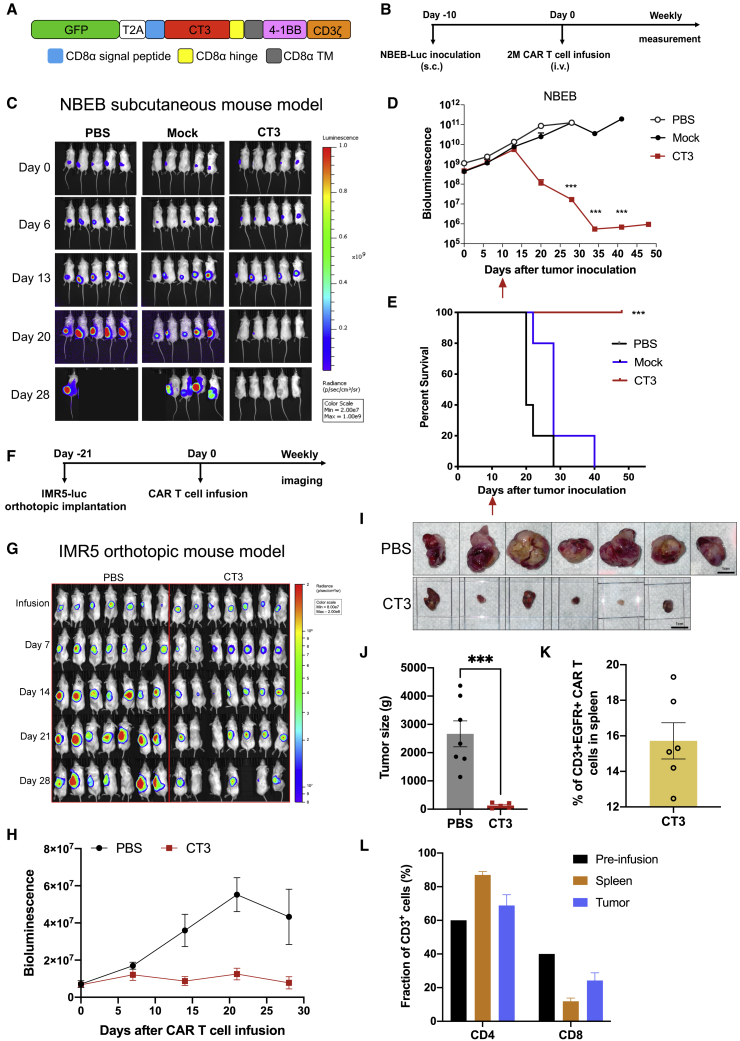
CT3 CAR T cells regress GPC2^+^ xenograft tumors in mice (A) Schematic of a CT3 CAR construct that is used for the subcutaneous NBEB model. The CAR transgene was cloned into the pLenti 6.3 vector. (B) Experimental schematic of the NBEB subcutaneous (s.c.) xenograft mouse model; 10M NBEB-mCherry-Luc were resuspended in Matrigel and s.c. injected into NSG mice. NBEB tumor-bearing mice were i.v. injected with 2M mock T cells or CT3 CAR T cells at day 10 post-tumor inoculation. (C) CT3 CAR T cells mediated complete regression of NBEB xenograft tumors. (D) Tumor bioluminescence of mice shown in (C). The red arrow indicates the time point of treatment. (E) Kaplan-Meier survival curve reveals that all of the mice in the CT3 CAR group shown in Figure (C)7C survived at the end of the study. (F) Experimental schematic of the IMR5 orthotopic xenograft mouse model. IMR5-Luc cells were injected through the left adrenal fat pad into the adrenal gland and 5M CT3 CAR T cells were intravenously infused at day 21 after tumor implantation. (G) CT3 CAR T cells regress tumor growth in mice. One mouse in the CT3 CAR group lost substantial weight and was pale. Other cage mates were unaffected. (H) Tumor bioluminescence in mice treated with CT3 CAR T cells in (G). (I) The sizes of tumors in mice from untreated and CT3 CAR groups at the end of the study. (J) The tumor weights in the CT3 CAR group are significantly smaller than the tumors in the untreated group. (K) CAR T cells were detected in spleens from the mice receiving CT3 CAR T cells. CD3^+^hEGFRt^+^ cells are considered CT3 CAR T cells. (L) The percentage of CD4^+^ and CD8^+^ T cells are analyzed in pre-infusion CAR T cells and spleens/tumors tissues from mice treated with CT3 CAR T cells. Values represent means ± SEMs. ∗∗∗p < 0.001.

We then tested CT3 CAR T cells in another NB xenograft mouse model. High GPC2-expressing NBEB cells (NBEB-luc) were subcutaneously (s.c.) inoculated into NSG mice. When the average BLI of the tumors reached 5 × 10^9^ after 10 days of inoculation, mice were given 2M mock T cells or CT3 CAR T cells once (Figures 7A and 7B). As shown in Figures 7C and 7D, all of the mice that received CT3 CAR T cells showed complete and sustained tumor regression; in other words, the tumor burden was undetectable by BLI over 5 weeks post-infusion. All of the mice given the treatment survived at the end of this study (Figure 7E).

Finally, we examined the antitumor activity of CT3 CAR T cells in an orthotopic NB mouse model. IMR5-luc cells were injected into the left adrenal fat pad into the adrenal gland and CT3 CAR T cells were i.v. infused at day 21 after tumor implantation, when tumors showed BLI consistent with the formation of an established tumor (Figure 7F). CT3 CAR T cells led to a reduction in tumor size and suppressed tumor growth compared with the untreated group (Figures 7G and 7H). At the end of this study, the CT3 CAR group showed very small residual tumors, whereas all of the mice in the untreated group carried large tumors (p = 0.0002, Figures 7I and 7J). Among the CD3^+^ T cells in the spleen of treated mice, 12%–20% expressed the CT3 CAR (Figure 7K). CT3 CAR T cells recovered from mice were predominantly CD4^+^ T cells with a CD4:CD8 ratio of 7.2 in splenic T cells compared to 1.5 before infusion (Figure 7L). In these different NB mouse models, we demonstrated that CT3 CAR T cells persist and drive the regression of NB tumors.

## Discussion

In the present study, we developed a strategy to identify tumor-associated exons (exons 3 and 7–10) in GPC2 by RNA-seq analysis and determined that exon 3 is rare in normal tissues but prominently expressed in cancer (NB). We also discovered CT3, a high-affinity mAb, which recognizes tumor-associated exons 3 and 10. The structure complex of CT3 and GPC2 showing the interaction of CT3 and exons 3 and 10 of GPC3 was visualized by EM. CT3 detected strong GPC2 expression in 95% of NB cases and had no detectable reactivity in any normal human tissues (except testis), including all of the major vital organs. Finally, we demonstrated that treatment with CT3 CAR T cells led to the regression of NB xenografts and resulted in the long-term persistence of CAR T cells in mice.

The role of isoforms in tumor targeting remains elusive. Some of these have been found to be enriched in tumors.^20^ In the present study, we found two of the GPC2 isoforms that encode proteins: *GPC2-201* with exons 1–10 and *GPC2-203* with exons 1–2 and 4–6. Since *GPC2-201* encodes the longest known isoform, and is predominantly expressed in NB.^12^^,^^13^ We hypothesized that exon 3 and C-terminal subunit exons 7–10 could be tumor-enriched epitopes for GPC2. The GPC2 exon expression analysis from the GTEx database verified that exon 3 has the lowest expression, followed by exons 7–10, in normal human tissues compared with other exons, indicating it could be a target for a potential exon-specific targeted therapy, to improve the discrimination of tumors from normal tissues. To test this strategy, we isolated the CT3 antibody that binds to both exons 3 and 10 of GPC2 and confirmed by EM structure of the GPC2/CT3 Fab complex. The structural analysis validates that CT3 interacts with the exon 3-encoded region of GPC2, in addition to exon 10. Future studies will be needed to reveal the structure complex in atomic detail.

Amplification of the *MYCN* oncogene is the most robust genetic factor correlated with poor clinical outcome in NB and can be found in :16%–20% of NB cases (and up to 40% in high-risk tumors).^32^ GPC2 expression is observed in nearly 90% of *MYCN* amplified NB cell lines (Figure 4B), together with the potent efficacy of CT3 CAR T cells in regressing xenograft tumors (IMR5 and NBEB) with *MYCN* amplification; therefore, we identify GPC2 as a potential immunotherapeutic target against *MYCN*-amplified NB. Future studies are needed to determine the antitumor efficacy of CT3 CAR T cells against *MYCN*-amplified PDXs. Moreover, GPC2 was detected in 33% *MYCN* non-amplified NB cell lines (Figure S3B). CT3 CAR T cells against *MYCN* non-amplified NB models should be tested in the future. Lastly, we have not analyzed GPC2 expression in NB cells with low levels of *MYCN* amplification, which also have poor overall survival when gain of the *MYCN* gene locus on the short arm of chromosome 2 occurs.^32^ It will be important to evaluate GPC2 levels in NB with various levels of *MYCN* amplification.

It has been reported recently that vector integration within the host *TET2* and *CBL* genes is associated with complete remission in one B cell leukemia patient treated with CD19 and another one treated with CD22 CAR T cells,^33^^,^^34^ suggesting that it is important to investigate clonal expansion in responders. In the present study, we analyzed the integration sites of the CT3 CAR in multiple mice from weeks 1 to 5 post-infusion. The 13 genes at integration sites enriched in responders at week 5 were commonly found to be involved in cell-signaling and chromatin modification pathways, consistent with a recent report that linked integration site distribution of the CD19 CAR with treatment outcomes.^35^ In particular, *PLCB1* and *BRD1* are two integrated genes that showed enrichment in more than half of responders (Figure 6E). The *BRD1* gene encodes BRD1 protein, which stimulates the acetylation of histones H3 and H4. It has been shown that BRD1 localizes at known enhancers in the CD8 gene, is responsible for the acetylation of H3, and is potentially crucial for efficient activation of CD8 expression.^36^ The shared integrated genes were observed in different tissues on the same mouse, likely due to the expansion of selected clones (Figure S7D). As vector integration can be associated with altered T cell differentiation, thereby increasing CAR T cell functionality,^33^ we examined the vector integration in defined subsets of pre-infused CT3 CAR T cells. A total of 22 integrated genes that were identified from mouse studies (Tables S5 and S6 occurred in these subsets (Table S7. Moreover, those genes have more accessibility in T_SCM_ and T_EMRA_ cells (Figure S6G). Several genes, including *GRB2*, *GIMAP7*, and *CTCF* have been reported to be important for T cell development and differentiation.^37^, ^38^, ^39^ For instance, *Grb2*-deficient T cells show defects in T cell development and increased T helper 1 (Th1) and Th17 cell differentiation capacities in mice.^39^ Lastly, we noticed one shared integrated gene in the current CT3 CAR T cells targeting GPC2, *PTPRA*, which was also found in the CAR (hYP7) T cells targeting GPC3 in hepatocellular carcinoma from our previous study,^19^ although at different integration sites. The protein encoded by *PTPRA* positively regulates T cell receptor (TCR) signaling by dephosphorylation of the inhibitory site of LCK and FYN kinases.^40^ Future studies are needed to investigate whether the disruption of regulatory genes at the site of vector integration could be responsible for the increased functionality of CAR T cells. Such studies may lead to the identification of “universal” regulators for CAR T cells.

In conclusion, we report an antibody (CT3) that recognizes tumor-associated exons on GPC2. CT3 CAR T cell therapy induces the sustained regression of NB in mouse models. Targeting tumor-associated exons described here may be a useful strategy for developing potent and safe CAR T cells and other therapeutics to treat solid tumors.

### Limitations of the study

Using recombinant proteins and peptides, we found that the CT3 antibody bound both exon 3 and 10 fragments of GPC2. We also used negative stain EM to visualize the structure of the GPC2:CT3 Fab complex, with the final reconstitution resolution of 21 Å, and showed that CT3 interacted with epitopes from both exons 3 and 10 of GPC2. However, high-resolution approaches such as cryo-EM are needed to more accurately reveal the CT3 epitope and its binding residues in atomic detail. We relied on an adult normal tissue microarray to show the restricted GPC2 expression in normal tissues. As NB most commonly affects children at age 5 or younger, it would be more clinically relevant to analyze GPC2 expression in normal tissues from infants and young children. IMR5 and NBEB NB xenograft mouse models were used for this study. Testing CT3 CAR T cells in other animal models, especially NB PDX models, would enable the evaluation of CT3 CAR T cell activity against more heterogenous tumors. These experiments will be pursued to further substantiate the results reported here.

## STAR★Methods

### Key resources table

**Table:** 

REAGENT or RESOURCE	SOURCE	IDENTIFIER
**Antibodies**		

CT1	This paper	N/A
CT2	This paper	N/A
CT3	This paper	N/A
CT4	This paper	N/A
CT5	This paper	N/A
CT6	This paper	N/A
CT7	This paper	N/A
CT8	This paper	N/A
CT9	This paper	N/A
CT10	This paper	N/A
Erbitux (anti-human EGFR antibody)	NIH Pharmacy	N/A
APC-H7 anti-human CD3 antibody	BD Biosciences	560176
BV786 anti-human CD3 antibody	Biolegend	317329
APC-H7 anti-human CD4 antibody	BD Biosciences	560158
APC anti-human CD4 Antibody	Biolegend	317415
APC-H7 anti-human CD8 antibody	BD Biosciences	560179
Alexa Fluor 488 anti-human CD8 antibody	Biolegend	344716
PerCP/Cyanine5.5 anti-human CD8 Antibody	Biolegend	301031
PE mouse anti-human CD25 antibody	Thermo Fisher Scientific	12-0259-42
PE mouse anti-human CD27 antibody	Thermo Fisher Scientific	12-0279-42
PE mouse anti-human CD127 antibody	Thermo Fisher Scientific	12-1278-42
PE mouse anti-human PD-1 antibody	Thermo Fisher Scientific	12-2799-42
PE mouse anti-human TIM-3 antibody	Thermo Fisher Scientific	12-3109-42
PE mouse anti-human LAG-3 antibody	Thermo Fisher Scientific	12-2239-42
PE Annexin V Apoptosis Detection Kit with 7-AAD	Biolegend	640934
BV421 mouse anti-human CD45RA antibody	BD Biosciences	562885
APC mouse anti-human CD62L antibody	BD Biosciences	559772
PE anti-human CD95 (Fas) Antibody	Biolegend	305608
goat-anti-mouse IgG conjugated with PE	Jackson ImmunoResearch	115-116-071
goat-anti-human IgG conjugated with PE	Jackson ImmunoResearch	109-116-170
goat-anti-human IgG conjugated with APC	Jackson ImmunoResearch	109-136-170
goat-anti-human IgG conjugated with Alexa Fluor 488	Jackson ImmunoResearch	109-546-170
goat-anti-mouse IgG conjugated with horseradish peroxidase (HRP)	Jackson ImmunoResearch	115-036-071
N-Myc	Santa Cruz Biotechnology	sc-53993
GAPDH	Cell Signaling Technology	2118

**Bacterial and virus strains**		

one shot Stbl3 chemically competent *E. coli*	Thermo Fisher Scientific	C737303

**Biological samples**		

human neuroblastoma tissue microarray	US Biomax, Inc.	NB642
human medulloblastoma tissue microarray	US Biomax, Inc.	BC17012
human retinoblastoma tissue microarray	US Biomax, Inc.	BC35111
human normal tissue microarray	US Biomax, Inc.	FDA999I
human peripheral blood mononuclear cells (PBMCs)	Oklahoma Blood Institute	N/A
SJNBL012407 PDX	St. Jude Children’s Research Hospital	N/A

**Chemicals, peptides, and recombinant proteins**		

GPC2 C-terminal peptide	This paper	N/A
GPC2 exon 3 18 amino acid peptides	This paper	N/A
recombinant human GPC2 protein	R&D systems, Inc.	2304-GP
recombinant mouse GPC2 protein	R&D systems, Inc.	2355-GP
recombinant human GPC1 protein	R&D systems, Inc.	4519-GP
recombinant human GPC3 protein	R&D systems, Inc.	2119-GP
recombinant human GPC4 protein	R&D systems, Inc.	9195-GP
recombinant human GPC5 protein	R&D systems, Inc.	2607-G5
recombinant human GPC6 protein	R&D systems, Inc.	2845-GP
human GPC2 exon3-hFc protein	This paper	N/A
Ni-NTA Biosensor	FortéBio	18-5101
Ficoll-Paque PLUS density gradient media	Cytiva	17-1440-03
human IL-2	NIH Pharmacy	N/A
D-Luciferin	PerkinElmer, Inc.	122799

**Critical commercial assays**		

Pierce Fab preparation kit	Thermo Fisher Scientific	44985
CalFectin mammalian cell transfection reagent	SignaGen Laboratories	SL100478
Lenti-X concentrator	Takara Bio	631232
Dynabeads human T-activator CD3/CD28 for T cell expansion and activation	Thermo Fisher Scientific	11132D
Luciferase assay system	Promega	E1501
DNeasy blood & tissue kit	QIAGEN	69506
Tumor dissociation kit, mouse	Miltenyi Biotec	130-096-730

**Deposited data**		

RNaseq of orthotopic PDXs from pediatric solid tumors	St. Jude Children’s Research Hospital	https://www.ebi.ac.uk/ega/datasets/EGAD00001003433
CT3 Fab/GPC2 complex EM maps	This paper	wwPDB deposition ID (D_1000256119)

**Experimental models: Cell lines**		

293T	ATCC	CRL-3216
A431	ATCC	CRL-1555
NBEB	NCI Pediatric Oncology Branch	N/A
IMR5	NCI Pediatric Oncology Branch	N/A
SKNAS	NCI Pediatric Oncology Branch	N/A
IMR32	NCI Pediatric Oncology Branch	N/A
Kelly	NCI Pediatric Oncology Branch	N/A
KCNR	NCI Pediatric Oncology Branch	N/A
LAN5	NCI Pediatric Oncology Branch	N/A
BE(2)C	NCI Pediatric Oncology Branch	N/A
NGP	NCI Pediatric Oncology Branch	N/A
LAN6	NCI Pediatric Oncology Branch	N/A
NBLS	NCI Pediatric Oncology Branch	N/A
SHIN	NCI Pediatric Oncology Branch	N/A
SKNFI	NCI Pediatric Oncology Branch	N/A
SKNMM	NCI Pediatric Oncology Branch	N/A
CHLA9	NCI Pediatric Oncology Branch	N/A
SHEP	NCI Pediatric Oncology Branch	N/A
SY5Y	NCI Pediatric Oncology Branch	N/A
HeLa	NCI Pediatric Oncology Branch	N/A
ARPE-19	NCI Pediatric Oncology Branch	N/A
A431 overexpressing GPC2 (G10)	This paper	N/A
IMR5 overexpressing GPC2 (F8)	This paper	N/A
IMR5-GFP and luciferase (Luc)	This paper	N/A
NBEB-mCherry and Luc	This paper	N/A
PDX-Luc	This paper	N/A
GPC2 knockout (KO)-IMR5	This paper	N/A
GPC2 KO-IMR5 expressing mCherry and Luc	This paper	N/A

**Experimental models: Organisms/strains**		

NOD-*scid IL2rg*^*null*^ (NSG) mice	NCI CCR Animal Resource Program/NCI Biological Testing Branch	N/A

**Oligonucleotides**		

GPC2 sgRNA sequence: GGACCAGGACCGGGACACAG	^12^	N/A
ddPCR CAR vector forward primer: GCAGTAGTCGCCGTGAAC	^19^	N/A
ddPCR CAR vector reverse primer: TCACCAGGAGAAGCATGGTGG	^19^	N/A
ddPCR MKL2 forward primer: AGATCAGAAGGGTGAGAAGAATG	^19^	N/A
ddPCR MKL2 reverse primer: GGATGGTCTGGTAGTTGTAGTG	^19^	N/A
CAR vector integration analysis first PCR 3′LTR: CAAGATGGGATCAATTCACCA	^19^	N/A
CAR vector integration analysis first PCR linker: GTAATACGACTCACTATAGGGC	^19^	N/A
CAR vector integration analysis nest 3′LTRnest: CCCTTTTAGTCAGTGTGGAAAATC	^19^	N/A
CAR vector integration analysis Nest PCR linker: AGGGCTCCGCTTAAGGGAC	^19^	N/A

**Recombinant DNA**		

CT3 4-1BB CAR (pMH303)	This paper	N/A
GD2 4-1BB CAR (pMH389)	This paper	N/A
psPAX2	Addgene	12260
pMD2.G	Addgene	12259
LentiCRISPRv2	Addgene	52961

**Software and algorithms**		

FlowJo 10.0	FlowJo, LLC	https://www.flowjo.com
Prism 9.0.0	Graphpad	https://www.graphpad.com
SnapGene 5.2.4	SnapGene	https://www.snapgene.com
Octet software 8.2	FortéBio	https://www.fortebio.com
SerialEM	^41^	https://bio3d.colorado.edu/SerialEM/
RELION 3.0.8	^42^	https://github.com/3dem/relion/releases/tag/3.0.8
STAR	^43^	https://github.com/alexdobin/STAR
RSEM	^44^	https://github.com/deweylab/RSEM
RTCA 2.0	Agilent	https://www.agilent.com
Aperio ImageScope 12.3.3	Leica Biosystems	https://www.leicabiosystems.com

### Resource availability

#### Lead contact

Requests for further information and reagents should be directed to and will be fulfilled by the Lead Contact, Mitchell Ho (homi@mail.nih.gov).

#### Materials availability

Plasmids and antibodies generated in this study can be made available under appropriate materials transfer agreement. No other unique reagents were generated.

#### Data and code availability

The RNaseq data of orthotopic PDXs of pediatric solid tumors are available: https://ega-archive.org/ega/datasets/EGAD00001003433

The CT3 Fab/GPC2 complex EM maps generated during this study are available at wwPDB with Deposition ID (D_1000256119): https://deposit-2.wwpdb.org/deposition/

### Experimental model and subject details

#### Cell lines

The A431 (epidermal carcinoma) and 293T cell lines were purchased from the American Type Culture Collection (ATCC). F8 is a transfected IMR5 cell line, and G10 is a transfected A431 cell line stably overexpressing human GPC2. GPC2 knockout (KO)-IMR5 cells were generated using CRISPR/Cas9 technology as described previously.^12^ A431, G10, NBEB, IMR5, F8, GPC2 KO-IMR5, SKNAS, NB PDX-derived cell lines were transduced with lentiviruses expressing firefly luciferase. All neuroblastoma cell lines (NBEB, IMR5, F8, GPC2 KO-IMR5, SKNAS, IMR32, Kelly, KCNR, LAN5, BE(2)C, NGP, LAN6, NBLS, SHIN, SK-N-FI, SK-N-MM, CHLA-9, SHEP, SY5Y) were cultured with RPMI 1640 supplemented with 10% FBS, 1% L-glutamine, and 1% penicillin–streptomycin at 37°C in a humidified atmosphere with 5% CO_2_. The 293T, HeLa, ARPE-19, G10 and A431 cells were cultured in DMEM supplemented with 10% FBS, 1% L-glutamine, and 1% penicillin–streptomycin at 37°C in a humidified atmosphere with 5% CO_2_. All cell lines were authenticated by morphology and growth rate and were mycoplasma free.

#### Primary cell culture

Whole blood was collected from healthy donors under the Oklahoma Blood Institute Institutional Review Board approval. Peripheral blood mononuclear cells (PBMCs) were isolated from the blood of healthy donors using Ficoll (Cytiva) according to manufacturer’s instructions. PBMCs were cultured with RPMI 1640 supplemented with 10% FBS, 1% L-glutamine, and 1% penicillin–streptomycin at 37°C in a humidified atmosphere with 5% CO_2_.

#### Neuroblastoma (NB) patient-derived xenograft (PDX)

PDX used in this study were obtained from Childhood Solid Tumor Network at St. Jude Children’s Research Hospital and have been propagated in NSG mice for 1–2 passages at Pediatric Oncology Branch, NCI, NIH. The St. Jude numbers of the PDX is SJNBL012407.

In order to generate a luciferase-expressing PDX-derived NB cell line, we transduced the PDX cells with CMV-Firefly luciferase lentivirus (Cellomics), and then the luciferase-expressing cells were selected and cultured in RPMI 1640 supplemented with 0.5 μg/ml puromycin, 10% FBS, 1% L-glutamine, and 1% penicillin–streptomycin at 37°C in a humidified atmosphere with 5% CO_2_.

#### Animals

5-week-old female NSG mice (NCI CCR Animal Resource Program/NCI Biological Testing Branch) were housed and treated under the protocol (LMB-059) approved by the Institutional Animal Care and Use Committee at the NIH.

#### Metastatic IMR5 model

For the 1^st^ metastatic IMR5 model, 7 million IMR5-luc tumor cells were i.v. injected into mice. After tumor establishment, the mice were randomized into four groups and separately injected i.v. via tail vein with various doses of CAR T cells once as follows: (a) 2.5 million untransduced T cells (Mock), (b) 2.5 million CT3 CAR T cells, (c) 5 million CT3 CAR T cells, and (d) 10 million CT3 CAR T cells. In the repeated study, 7 million IMR5-luc tumor cells were i.v. injected into mice. Mice with established tumors were randomly allocated into two groups and i.v. infused once with 10 million mock T cells and CT3 CAR T cells. For the study of CAR T cells distribution in various mouse tissues, IMR5 tumor-bearing mice were i.v. infused once with 5 million CT3 CAR T cells and control CAR T cells targeting GPC3. Tumors were measured by total bioluminescent flux using a Xenogen IVIS Lumina (PerkinElmer). Living Image software was used to analyze the bioluminescence signal flux for each mouse as photons/s/cm^2^/sr. Mice were euthanized when mice showed any sign of sickness or bioluminescence signal reached 1 × 10^10^. At the time of animal sacrifice, cells were collected for flow cytometry or *ex vivo* analysis. Dissociation of tumor/spleen tissues was performed using Miltenyi Biotec tumor dissociation kit. Isolated T cells from above tissues were then stained for CD3 and EGFR expression.

#### Subcuteneous (s.c.) NBEB model

10 million NBEB-mCherry-Luc tumor cells were resuspended in Matrigel and s.c. injected into mice. After tumor establishment, the mice were randomized into three groups and i.v. infused with 2 million mock or CT3 CAR T cells once. Tumor size was measured using a Xenogen IVIS Lumina every week. Living Image software was used to analyze the bioluminescence signal flux for each mouse as photons/s/cm^2^/sr. Mice were euthanized when mice showed any sign of sickness or bioluminescence signal reached 1 × 10^11^.

#### Orthotopic IMR5 model

0.25 million IMR5-luc tumor cells were injected into the left adrenal fat pad into the adrenal gland. 5 million CT3 CAR T cells were intravenously infused at day 21 after tumor implantation. Tumors size were measured using a Xenogen IVIS Lumina every week. Living Image software was used to analyze the bioluminescence signal flux for each mouse as photons/s/cm^2^/sr. At the end of the study, tumor dimensions were determined using calipers, and tumor volume (mm^3^) was calculated by the formula V = 1/2 ab^2^, where a and b represent tumor length and width, respectively. At the time of animal sacrifice, cells were collected for flow cytometry or *ex vivo* analysis. Dissociation of tumor/spleen tissues was performed using Miltenyi Biotec tumor dissociation kit. Isolated T cells from above tissues were then stained for CD3, CD4, CD8, and EGFR expression.

### Method details

#### Isolation of anti-GPC2 mouse mAbs

The isolation of mouse mAbs against glypican was described previously.^45^ Briefly, the process includes peptide synthesis, immunization, spleen cell fusion, hybridoma selection and expansion. The C-terminal peptide consisting of 50 residues was synthesized by GenScript. Hybridoma cells were screened via ELISA, flow cytometry and immunohistochemistry. The CT3 clone, which displayed the highest affinity and most specific binding, was chosen for purification and further characterization.

#### ELISA

Mouse hybridoma supernatant containing 1 μg/ml of each mAb was incubated with plates coated with recombinant human GPC2, GPC1, and GPC3. The CT3 antibody at 1 μg/ml was incubated with human GPC1 through GPC6 and mouse GPC2 proteins purchased from R&D Systems. Exon 3 of GPC2 was fused with human Fc and then cloned into the pVRC8400 expression plasmid as previously described.^46^ Binding was detected with a goat anti-mouse IgG conjugated with horseradish peroxidase (HRP) (Jackson ImmunoResearch).

Twelve peptides comprising exon 3 of GPC2 were synthesized by Genscript. Each peptide is 18 amino acids long and has 9 overlapped amino acids with adjacent peptide. The CT3 binding reactivity to each peptide was evaluated by ELISA. 5 μg/ml peptides in PBS were used to coat the plates overnight. 1 μg/ml of CT3 was added to the assay wells and the binding was detected with a goat anti-mouse IgG conjugated with HRP. For conformational epitope mapping of CT3 in exon 3, the adjacent two peptides at 5 μg/ml were then mixed together at room temperature for 1 hour. Then, the 2 × 2 matrix mixture was made by mixing the prepared peptide mixtures at room temperature for 1 hour. The final mixtures at 5 μg/ml were used to coat the plates. 1 μg/ml of CT3 was added to the assay wells and the binding was detected with a goat anti-mouse IgG conjugated with HRP.

#### Flow cytometry

LAN5 neuroblastoma cells were incubated with mouse hybridoma supernatant containing 10 μg/ml of each mAb. Cell binding was then detected with a goat anti-mouse IgG conjugated with phycoerythrin (PE). For analysis of GPC2 expression on the cell surface, tumor cells were incubated with 10 μg/ml of CT3 mAb and detected with a goat anti-mouse IgG conjugated with allophycocyanin (APC). To measure lentiviral transduction efficiencies, CT3 CAR expression on T cells was detected with the anti-EGFR human monoclonal antibody cetuximab (Erbitux) and goat-anti-human IgG conjugated with PE. All secondary antibodies were purchased from Jackson ImmunoResearch. Data acquisition was performed using FACSCantoII (BD Biosciences) and analyzed using FlowJo software (Tree Star).

T cell activation and exhaustion were evaluated via APC-H7 CD4 (BD Bioscience); Alexa Fluor 488 CD8 (Biolegend); PE CD25, PE CD27, PE CD127, PE PD-1, PE TIM-3, PE LAG-3 (Thermo Fisher Scientific). CAR expression was detected with the anti-EGFR human monoclonal antibody cetuximab (Erbitux) and goat-anti-human IgG conjugated with APC (Jackson ImmunoResearch). T cell apoptosis was analyzed using an annexin V/7-AAD kit (Biolegend). All FACS plots representing CAR T cell data were conducted on gated CAR^+^ cells. For mock T cells, T cell population was used for analysis. Data acquisition was performed using SONY SA3800 (Sony Biotechnology) and analyzed using FloJo software (Tree Star).

T cell immunophenotyping was performed by surface staining with antibodies against the following antigens: APC-H7 CD4, APC-H7 CD8, BV421 CD45RA, APC CD62L (BD Bioscience); and PE CD95 (Biolegend). CAR expression was detected with Erbitux and goat-anti-human IgG conjugated with Alexa Fluor 488 (Jackson ImmunoResearch). All FACS plots representing CAR T cell data were conducted on gated CAR^+^ cells. For unstimulated PBMCs and mock T cells, T cell populations was used for analysis. Data acquisition was performed using SONY SA3800 (Sony Biotechnology) and analyzed using FloJo software (Tree Star).

Cell sorting of different memory T cell subsets was performed by surface staining with antibodies against the following antigens: APC-H7 CD3, BV421 CD45RA, and APC CD62L (BD Bioscience). CAR expression was detected with Erbitux and goat-anti-human IgG conjugated with Alexa Fluor 488. All FACS plots representing CAR T cell data were conducted on gated CAR^+^ cells. Cell sorting was done using BD FACSAria II. Genomic DNA of each sorted population was extracted and used for integration site analysis.

For T cells isolated from mouse tissues were evaluated via BV786 CD3, APC CD4, and PerCP/Cyanine5.5 CD8 (Biolegend). CAR expression was detected with Erbitux and goat-anti-human IgG conjugated with PE. Data acquisition was performed using FACSCantoII (BD Biosciences) and analyzed using FloJo software (Tree Star).

#### Immunohistochemistry

Normal tissue and pediatric tumor tissue microarrays were purchased from US Biomax. Normal tissue sections were stained with mouse hybridoma supernatant containing 1 μg/ml of the CT3 mAb. Neuroblastoma, medulloblastoma, and retinoblastoma sections were stained with 1 μg/ml CT3 mAb. The immunohistochemical staining was performed by Histoserv Inc.

#### Antibody binding assay

The binding kinetics of the CT3 mAb to GPC2 was determined using the Octet RED96 system (FortéBio). His-tagged GPC2 protein (R&D Systems, Inc) was immobilized onto Ni-NTA biosensors, which were subsequently used in association and dissociation measurements for a time window of 600 s and 1800 s, respectively. Data analysis was performed using the FortéBio analysis software.

#### Analysis of GPC2 exon expression

To understand the gene expression in specific normal tissues, GTEx collected the WGS, WES and RNA-seq data of 54 non-diseased tissues from 948 donors. In addition to the transcript and junction expression, it also reports exon expression, the median read counts per base, i.e., the median raw read counts normalized by exon length, for each exon.

#### Negative stain EM preparation of GPC2-CT3 Fab complex and data collection

The CT3 antigen-binding fragment (Fab) was prepared using a Fab preparation kit (Thermo Scientific). GPC2 protein was mixed with CT3 Fab at 1:1 molar ratio in PBS. A 3 μl aliquot containing ∼0.01 mg/ml of the sample was applied for 20 s onto a carbon-coated 200 Cu mesh grid (Electron Microscopy Sciences, Protochips, Inc.) that had been glow discharged at 30 mA for 30 s (Pelco easiGlow, Ted Pella, Inc.), then negatively stained with 0.7% (w/v) uranyl formate for 40 s. Data was collected using a Tecnai FEI T20 electron microscope operating at 200 kV, with an electron dose of ∼40 e-/Å2 and a magnification of 100,000 x that resulted in a pixel size of 2.19 Å at the specimen plane. Images were acquired with an Eagle 2kx2k CCD camera (FEI) using a nominal defocus of 1100 nm and the SerialEM software.^41^

#### Negative stain EM data processing and model building

Particles were selected from the micrographs, extracted, and a reference-free 2D class averages were obtained using RELION 3.0.8.^42^ After 2D sorting, particles were subject to 3D classification, requesting 6 classes, and starting with an initial model of the GPC2 unliganded and filtered to 60 Å resolution without imposing symmetry. A new set of particles were picked using a rather high threshold (> 0.9). 2D classifications were performed on the new set of particles, first with 50 classes, then with 20 classes. Bad particles were discarded after each 2D classification. 3D classifications were followed with either 5 or 3 classes. Bad particles were again discarded after this step. Lastly, the particles that contributed to the best 3D classification model were selected for 3D refinement. Final model was produced when the 3D refinements were converged. All the above procedures were carried out in RELION-3.0.8.

#### RNA sequencing

For the analysis of *GPC2* mRNA expression in neuroblastoma cell lines, Ribozero-selected RNA libraries were prepared for RNA sequencing on Illumina NextSeq500 according to the manufacture’s protocol (Illumina, San Diego, CA). Sequencer-generated bcl files were converted to fastq files using the bcl2fastq tool in *CASAVA* (Illumina, San Diego, CA) suite. Paired-end reads (75 bp) were assessed for quality using FastQC. Fastq files were then mapped to GRCH37 reference genome using the STAR/2.5.3a alignment algorithm^43^ and subsequently quantified by RSEM program^44^ based upon Ensembl GRCh37.75 gene annotation.

For the analysis of *GPC2* mRNA expression in orthotopic PDXs from pediatric solid tumors, host PDX reads were first filtered from human reads using Xengsort.^47^ The remaining reads were mapped to hg19 genome using STAR with 2-pass mode.^43^ The transcriptome BAM file was used to estimate the gene level abundances using RSEM.^44^ The UCSC annotation was used to calculate the read counts.^48^ The raw read counts for each gene were further normalized by the transcripts per million (TPM).^49^

#### Immunoblotting

Cells were lysed with ice-cold lysis buffer (Cell Signaling Technology), and clarified by centrifugation at 10,000 g for 10 minutes at 4°C. Protein concentration was measured using a Bicinchoninic acid assay (Pierce) following the manufacturer’s specifications. Cell lysates were loaded onto a 4%–20% SDS-PAGE gel for electrophoresis. CT3 was used to detect GPC2 expression. The anti-N-Myc antibody was purchased from Santa Cruz Biotechnology. The anti-GAPDH antibody was obtained from Cell Signaling Technology.

#### Generation of GPC2-specific CAR T cells

The CT3 variable regions were cloned using 5′RACE with modified primers and conducted as described previously.^50^^,^^51^ The single-chain variable fragment (scFv) of CT3 was subcloned into a 4-1BB-based CAR construct (pMH303). The hEGFRt was included for cell tracking and ablation. CT3 CAR lentiviruses were produced by co-transfecting with packaging plasmid psPAX2 and envelope plasmid pMD2.G into HEK293T cells using Calfectin (SignaGen) as described previously.^19^ PBMCs from healthy donors were stimulated for 24h using anti-CD3/anti-CD28 antibody-coated beads (Invitrogen) at a bead: cell ratio of 2:1 according to manufacturer’s instructions in the presence of IL-2. To track T cell numbers over time, viable cells were counted using trypan blue.

#### Luciferase-based cytolytic assay

The cytolytic activity of GPC2-targeted CAR T cells was determined using a luciferase-based assay as previously described.^12^^,^^19^ In brief, CT3 CAR T cells and mock T cells were cocultured with luciferase-expressing GPC2-positive cells (IMR5), GPC2-overexpressing cells (F8 derived from IMR5, G10 derived from A431), and GPC2-negative cells (GPC2 KO-IMR5, A431) at different ratios for 24 hours. The luciferase-expressing PDX (SJNBL012407)-derived neuroblastoma cells were co-cultured with CT3 CAR T cells and mock T cells at various times (24 hours, 48 hours and 72 hours). To analyze function of isolated CAR T cells from mouse tissues, T cells were co-cultured with IMR5 or GPC2 KO-IMR5 cells, and the cytolytic activities were measured after 24 hours of co-culture. The luciferase activity was measured using the luciferase assay system (Promega) on Victor (PerkinElmer).

#### xCELLigence real-time cell analysis (RTCA)

Human neuroblastoma cell lines NBEB and SKNAS were seeded into 96 well E-Plates at 1 × 10^5^ cells per well. Then the E-Plate 96 was placed in the impedance-based real-time cell analysis (RTCA) xCELLigence system (Acea Biosciences) for 5 hours to allow cell attachment and proliferation. The mock T cells or CT3 CAR T cells were added to the target cells in 1:1 effector to target ratio and the killing of the target cells was continuously measured for an additional 20 hours. Data were analyzed with RTCA Software 2.0. Cytolysis was normalized with impedance of target cells before adding effector T cells.

#### ddPCR

Tissues were homogenized using the Bullet Blender, and genomic DNA from cells was isolated using the DNeasy blood & tissue kit (QIAGEN). Digital PCR was performed on a QX200 droplet digital PCR system (Bio-Rad) according to the manufacturer’s instructions. CAR vector specific primers and probe were multiplexed with either a human (myocardin-like protein 2, MKL2) or mouse (Transferrin Receptor, Tfrc) reference gene assay described previously.^19^

#### Integration site analysis

CAR lentivector integration site analysis was performed using linker-mediated PCR adapted from a procedure described previously for measuring viral infection in HIV patients.^52^ Briefly, sample DNA is randomly sheared, end-repaired, and ligated to a linker. The integration site is amplified with one primer specific to the lentivector LTR and another primer specific to the linker. The amplified product is subjected to high-throughput Illumina Sequencing. Integration sites in the sample are identified and quantified for further analysis. The primer sequences designed for the present study were described previously.^19^

### Quantification and statistical analysis

#### Statistical analysis

All experiments were repeated a minimum of three times to ensure reproducibility of results. All statistical analyses were performed using GraphPad Prism, and are presented as mean ± SEM. Results were analyzed using 2-tailed unpaired Student’s t test. A P value of < 0.05 was considered statistically significant.

## Supplemental information

Document S1. Tables S1–S7 and Figures S1–S7

Document S2. Article plus supplemental information
