# Supplementary material for: CAR T cells targeting tumor-associated exons of glypican 2 regress neuroblastoma in mice
Source: Cell Rep Med. 2021 Jun 1;2(6):100297. doi: 10.1016/j.xcrm.2021.100297 (PMC8233664; doi:10.1016/j.xcrm.2021.100297)
Supplement: Document S1. Tables S1–S7 and Figures S1–S7 [file mmc1.pdf]

Cell Reports Medicine, Volume *2*

## Supplemental information

# CAR T cells targeting tumor-associated exons of glypican 2 regress neuroblastoma in mice

**Nan Li, Madeline B. Torres, Madeline R. Spetz, Ruixue Wang, Luyi Peng, Meijie Tian, Christopher M. Dower, Rosa Nguyen, Ming Sun, Chin-Hsien Tai, Natalia de Val, Raul Cachau, Xiaolin Wu, Stephen M. Hewitt, Rosandra N. Kaplan, Javed Khan, Brad St Croix, Carol J. Thiele, and Mitchell Ho**

**Table S1. The detailed information of tissue specimens shown in Figure S1B. Related to Figure 1.**

| Organ (Anatomic Site) | Age | Sex | Pathology diagnosis | Type |
|---|---|---|---|---|
| Cerebrum | 2 | F | Gray matter tissue | Normal |
| Cerebellum | 24 | F | Cerebellum tissue | Normal |
| Hypophysis | 27 | F | Hypophysis tissue | Normal |
| Adrenal gland | 18 | F | Adrenal gland tissue | Normal |
| Ovary | 36 | F | Ovary tissue | Normal |
| Pancreas | 16 | F | Pancreas tissue | Normal |
| Prostate | 31 | M | Prostate tissue | Normal |
| Nerve | 31 | M | Peripheral nerve tissue | Normal |
| Parathyroid gland | 50 | M | Thyroid gland tissue | Normal |
| Thymus gland | 15 | F | Thymus gland tissue | Normal |
| Pleural | 19 | F | Pleural mesothelium tissue | Normal |
| Thyroid gland | 18 | F | Thyroid gland tissue | Normal |
| Breast | 21 | F | Breast tissue | Normal |
| Spleen | 21 | F | Spleen tissue | Normal |
| Uterine cervix | 46 | F | Adjacent normal cervix tissue | NAT |
| Eye | 47 | F | Cancer adjacent choroid tissue | NAT |
| Tonsil | 23 | M | Tonsil tissue | Normal |
| Small intestine | 30 | M | Small intestine tissue | Normal |
| Bone marrow | 21 | F | Bone marrow tissue | Normal |
| Lung | 24 | M | Lung tissue | Normal |
| Heart | 35 | M | Cardiac muscle tissue | Normal |
| Esophagus | 45 | M | Esophagus tissue | Normal |
| Striated muscle | 40 | M | Skeletal muscle tissue | Normal |
| Larynx | 62 | M | Submucosal gland tissue | NAT |
| Stomach | 29 | M | Stomach tissue | Normal |
| Uterus | 40 | F | Adjacent normal endometrium tissue | NAT |
| Colon | 29 | M | Colon tissue | Normal |
| Liver | 38 | M | Liver tissue | Normal |

| | | | | |
|---|---|---|---|---|
| Tongue | 15 | F | Salivary gland tissue | Normal |
| Kidney | 16 | M | Kidney tissue | Normal |
| Skin | 50 | F | Skin tissue | Normal |
| Testis | 30 | M | Testis tissue | Normal |

**Table S2. The identified GPC2 mRNA transcripts. Related to Figure 2.**

| Name | Transcript ID | BP | Exons | Protein | Biotype | UniProt | RefSeq |
|---|---|---|---|---|---|---|---|
| ***GPC2-201*** | ENST00000292377.4 | 2532 | 1-10 | 579aa | Protein coding | Q8N158 | NM_152742 NP_689955 |
| *GPC2-202* | ENST00000471717.1 | 2129 | | No protein | Retained intron | - | - |
| ***GPC2-203*** | ENST00000480087.5 | 767 | 1-2, 4-6 | 134aa | Nonsense mediated decay | A0A0J9YXG7 | - |
| *GPC2-204* | ENST00000486702.1 | 787 | | No protein | Retained intron | - | - |
| *GPC2-205* | ENST00000490629.5 | 798 | | No protein | Processed transcript | - | - |

**Table S3. The amino acid sequence of GPC2 exon 3 and the comprising 12 peptides. Related to Figure 3.** Each peptide is 18 amino acids long and has 9 overlapped amino acids with adjacent peptide. The predicted epitope containing region in exon 3 is highlighted with red. The overlapped sequences in peptides 10 and 11, peptides 14 and 15 are bolded.

| Assigned name | Sequence |
|---|---|
| GPC2 Exon 3 | EFFLEMLSVAQHSLTQLFSHSYGRLYAQHALIFNGLFSR **LRDFYGESGEGLDDT**LADFWAQLLERVFPLLHPQYSF PPDYLLCLSRLASSTDGSLQPFGDSPRRLRLQ |
| Peptide 10 | RKFDEFFLE**MLSVAQHSL** |
| Peptide 11 | **MLSVAQHSL**TQLFSHSYG |
| Peptide 12 | TQLFSHSYGRLYAQHALI |
| Peptide 13 | RLYAQHALIFNGLFSRLR |
| Peptide 14 | FNGLFSRLR**DFYGESGEG** |
| Peptide 15 | **DFYGESGEG**LDDTLADFW |
| Peptide 16 | LDDTLADFWAQLLERVFP |
| Peptide 17 | AQLLERVFPLLHPQYSFP |
| Peptide 18 | LLHPQYSFPPDYLLCLSR |

| | |
|---|---|
| Peptide 19 | PDYLLCLSRLASSTDGSL |
| Peptide 20 | LASSTDGSLQPFGDSPRR |
| Peptide 21 | QPFGDSPRRLRLQITRTL |

**Table S4. The detailed information of tissue specimens shown in Figure S4. Related to Figure 4.**

| Panel | Position | Age | Sex | Organ/ Anatomic Site | Pathology diagnosis | Type |
|---|---|---|---|---|---|---|
| A | A1 | 3 | F | Retroperitoneum | Neuroblastoma | Malignant |
| | A2 | 8 | F | Retroperitoneum | Neuroblastoma | Malignant |
| | A3 | 1 | F | Retroperitoneum | Neuroblastoma | Malignant |
| | A4 | 7 | M | Retroperitoneum | Neuroblastoma | Malignant |
| | A5 | 4 | F | Retroperitoneum | Neuroblastoma | Malignant |
| | B1 | 6 | M | Retroperitoneum | Neuroblastoma | Malignant |
| | B2 | 3 | F | Retroperitoneum | Neuroblastoma | Malignant |
| | B3 | 4 | F | Retroperitoneum | Neuroblastoma | Malignant |
| | B4 | 1 | F | Retroperitoneum | Neuroblastoma | Malignant |
| | B5 | 2 | F | Retroperitoneum | Neuroblastoma | Malignant |
| | C1 | 5 | M | Retroperitoneum | Neuroblastoma | Malignant |
| | C2 | 4 | F | Retroperitoneum | Neuroblastoma | Malignant |
| | C3 | 8 Mon. | M | Retroperitoneum | Neuroblastoma | Malignant |
| | C4 | 1 | M | Retroperitoneum | Neuroblastoma | Malignant |
| | C5 | 4 | M | Retroperitoneum | Neuroblastoma | Malignant |
| | D1 | 1 | F | Mediastinum | Neuroblastoma | Malignant |
| | D2 | 2 | F | Mediastinum | Neuroblastoma | Malignant |
| | D3 | 6 | M | Adrenal gland | Neuroblastoma | Malignant |
| | D4 | 6 | M | Adrenal gland | Neuroblastoma (low differentiation type) | Malignant |
| | D5 | 5 | F | Adrenal gland | Neuroblastoma with necrosis (low differentiation type) | Malignant |
| | E1 | 31 | M | Nerve | Peripheral nerve tissue | Normal |
| | E2 | 36 | M | Nerve | Peripheral nerve tissue | Normal |
| | E3 | 33 | M | Nerve | Peripheral nerve tissue | Normal |
| | E4 | 25 | M | Nerve | Peripheral nerve tissue | Normal |

| | | | | | | |
|---|---|---|---|---|---|---|
| | E5 | 32 | M | Nerve | Peripheral nerve tissue | Normal |
| B | A1 | 8 | M | Cerebellum | Medulloblastoma | Malignant |
| | A2 | 16 | M | Cerebrum | Medulloblastoma | Malignant |
| | A3 | 14 | M | Cerebrum | Medulloblastoma | Malignant |
| | A4 | 9 | M | Cerebrum | Medulloblastoma | Malignant |
| | A5 | 7 | M | Cerebellum | Medulloblastoma | Malignant |
| | B1 | 12 | F | Cerebrum | Medulloblastoma | Malignant |
| | B2 | 8 | F | Cerebellum | Medulloblastoma | Malignant |
| | B3 | 14 | F | Cerebellum | Medulloblastoma of vermis cerebellum | Malignant |
| | B4 | 4 | F | Cerebrum | Medulloblastoma of vermis cerebellum | Malignant |
| | B5 | 6 | M | Cerebellum | Medulloblastoma of vermis cerebellum | Malignant |
| | C1 | 6 | F | Cerebellum | Medulloblastoma | Malignant |
| | C2 | 35 | M | Cerebrum | Normal cerebrum tissue | Normal |
| | C3 | 48 | M | Cerebrum | Normal cerebrum tissue | Normal |
| | C4 | 24 | F | Cerebellum | Normal cerebellum tissue | Normal |
| C | A1 | 15 | F | Eyeball | Retinoblastoma | Malignant |
| | A2 | 2 | F | Eye | Retinoblastoma | Malignant |
| | A3 | 11 | F | Eye | Retinoblastoma | Malignant |
| | A4 | 3 | M | Eye | Retinoblastoma | Malignant |
| | B1 | 2 | M | Eye | Retinoblastoma | Malignant |
| | B2 | 3 | M | Eye | Retinoblastoma | Malignant |
| | B3 | 2 | M | Eye | Retinoblastoma (choroid tissue) | Malignant |
| | B4 | 8 Mon. | M | Eye | Retinoblastoma with necrosis | Malignant |
| | C1 | 4 | M | Eye | Retinoblastoma (sparse) | Malignant |
| | C2 | 63 | M | Eye | Cancer adjacent normal retina and choroid tissue | NAT |
| | C3 | 1 | M | Eye | Cancer adjacent normal cornea tissue | NAT |
| | C4 | 2 | F | Eye | Cancer adjacent normal sclera and choroid tissue | NAT |

**Table S5. The shared integration sites in spleens from mice received CT3 CAR T cells from week 1 to week 5. Related to Figure 6.** The genes found in the memory T cell subsets shown in Table S5 are bolded and underlined.

| Gene | Integration site | Strand | Gene description |
|---|---|---|---|
| ***PLCB1*** | chr20:8839536 | - | Phospholipase C, β1 |
| ***STX5*** | chr11: 62578465 | + | Syntaxin 5 |
| ***GRB2*** | chr17: 73327158 | - | Growth factor receptor bound protein 2 |
| ***PACS1*** | chr11: 65884798 | + | Phosphofurin acidic cluster sorting protein 1 |
| *CLASRP* | chr19: 45550055 | - | CLK4-associating serine/arginine rich protein |
| ***KDM2A*** | chr11: 66971958 | - | Lysine demethylase 2A |
| ***IP6K2*** | chr3: 48743907 | - | Inositol hexakisphosphate kinase 2 |
| *SEPW1* | chr19: 48288693 | + | Selenoprotein W |
| *PITPNA* | chr17: 1447968 | - | Phosphatidylinositol transfer protein alpha |
| *BRD1* | chr22: 50213836 | - | Bromodomain-containing protein 1 |
| *BCL2L12* | chr4: 48388789 | + | BCL2 like 12 |
| ***NSD1*** | chr19: 57807300 | - | Nuclear receptor binding SET domain protein 1 |
| *BRIP1* | chr17: 59883538 | - | BRCA1 interacting protein C-terminal helicase 1 |
| *TBC1D20* | chr20: 427307 | + | TBC1 domain family member 20 |
| ***XRCC5*** | chr2: 216994979 | + | X-ray repair cross-complementing protein 5 |
| *BCLAF1* | chr6: 136596651 | - | BCL2 associated transcription factor 1 |

**Table S6. The shared integration sites in various tissues from mice infused with CT3 CAR T cells. Related to Figure 6.** The genes found in the memory T cell subsets shown in Table S5 are bolded and underlined.

| Gene | Integration site | Strand | Gene description |
|---|---|---|---|
| ***SEC16A*** | chr9: 139346426 | + | SEC16 homolog A, endoplasmic reticulum export factor |
| ***IP6K1*** | chr3: 49807052 | + | Inositol hexakisphosphate kinase 1 |
| *RBM4* | chr11: 66409814 | - | RNA binding motif protein 4 |
| ***GIMAP7*** | chr7: 150218051 | - | GTPase, IMAP family member 7 |
| *SNX12* | chrX: 70251385 | - | Sorting nexin 12 |
| ***NUMA1*** | chr11: 66971958 | + | Nuclear mitotic apparatus protein 1 |
| *KIF1B* | chr1: 10335597 | + | Kinesin family member 1B |
| *PDIA4* | chr7: 148715371 | + | Protein disulfide isomerase family A member 4 |

| | | | |
|---|---|---|---|
| *GFER* | chr16: 2036134 | + | Growth factor, augmenter of liver regeneration |
| ***ANKRD11*** | chr16: 89524020 | - | Ankyrin repeat domain 11 |
| *NT5C2* | chr10: 104940883 | - | 5'-Nucleotidase, cytosolic II |
| *UHRF1* | chr19: 4946341 | - | Ubiquitin like with PHD and ring finger domains 1 |
| *RIF1* | chr2: 152333245 | - | Replication timing regulatory factor 1 |
| *UBE2E3* | chr2: 182040950 | + | Ubiquitin conjugating enzyme E2 E3 |
| ***HORMAD2*** | chr22: 30520531 | - | HORMA domain containing 2 |
| *DUS1L* | chr17: 80026496 | - | Dihydrouridine synthase 1 like |
| *VPREB1* | chr22: 22493168 | + | V-Set pre-B cell surrogate light chain 1 |
| ***CTCF*** | chr16: 67612199 | + | CCCTC-binding factor |
| *YTHDC2* | chr5: 112883205 | + | YTH domain containing 2 |
| ***PPP6R2*** | chr22: 50827467 | + | Protein phosphatase 6 regulatory subunit 2 |
| ***LRBA*** | chr4: 151412815 | - | LPS responsive beige-like anchor protein |
| ***ARNT*** | chr1: 150857347 | - | Aryl hydrocarbon receptor nuclear translocator |
| ***POLRMT*** | chr19: 619348 | + | RNA polymerase mitochondrial |
| *C17orf70* | chr17: 79508608 | - | FA core complex associated protein 100 |
| *LGALS14* | chr19: 40201907 | + | galectin 14 |
| *ZMYND11* | chr10: 244271 | - | Zinc finger MYND-type containing 11 |
| ***CHD3*** | chr17: 7781348 | - | Chromodomain helicase DNA binding protein 3 |
| *C9orf86* | chr9: 139706625 | - | Rab-like protein 6 |
| ***PTPRA*** | chr20: 2930744 | + | Receptor-type tyrosine-protein phosphatase alpha |
| ***KIAA0753*** | chr17: 6531444 | + | KIAA0753 |

**Table S7. The 22 shared integrated genes identified from in vivo studies shown in Tables S5 and S6 were found in different cultured memory T cell subsets. Related to Figure 6.** The times of each integrated gene appeared were counted. NA: not applicable.

| Shared integrated genes from mouse studies | CT3 CAR T cells | | | |
|---|---|---|---|---|
| | $T_{cm}$ | $T_{scm}$ | $T_{emra}$ | $T_{em}$ |
| *PLCB1* | NA | NA | 1 | NA |
| *STX5* | NA | NA | 1 | NA |
| *GRB2* | NA | 1 | NA | NA |
| *PACS1* | 2 | 1 | 2 | 3 |
| *KDM2A* | NA | 2 | 1 | NA |
| *IP6K2* | NA | NA | 1 | NA |
| *NSD1* | NA | NA | NA | 2 |
| *XRCC5* | NA | NA | 1 | NA |
| *SEC16A* | 2 | NA | NA | 2 |
| *IP6K1* | NA | 1 | 1 | NA |
| *GIMAP7* | NA | 1 | 1 | NA |
| *NUMA1* | NA | 1 | 2 | NA |
| *ANKRD11* | NA | 1 | 1 | NA |
| *HORMAD2* | NA | 1 | NA | 1 |
| *CTCF* | 1 | 1 | 1 | NA |
| *PPP6R2* | NA | NA | 1 | NA |
| *LRBA* | 1 | NA | 1 | 1 |
| *ARNT* | NA | 1 | NA | NA |
| *POLRMT* | 1 | NA | NA | 1 |
| *CHD3* | 1 | 2 | 1 | NA |
| *PTPRA* | NA | NA | NA | 1 |
| *KIAA0753* | NA | 2 | NA | NA |

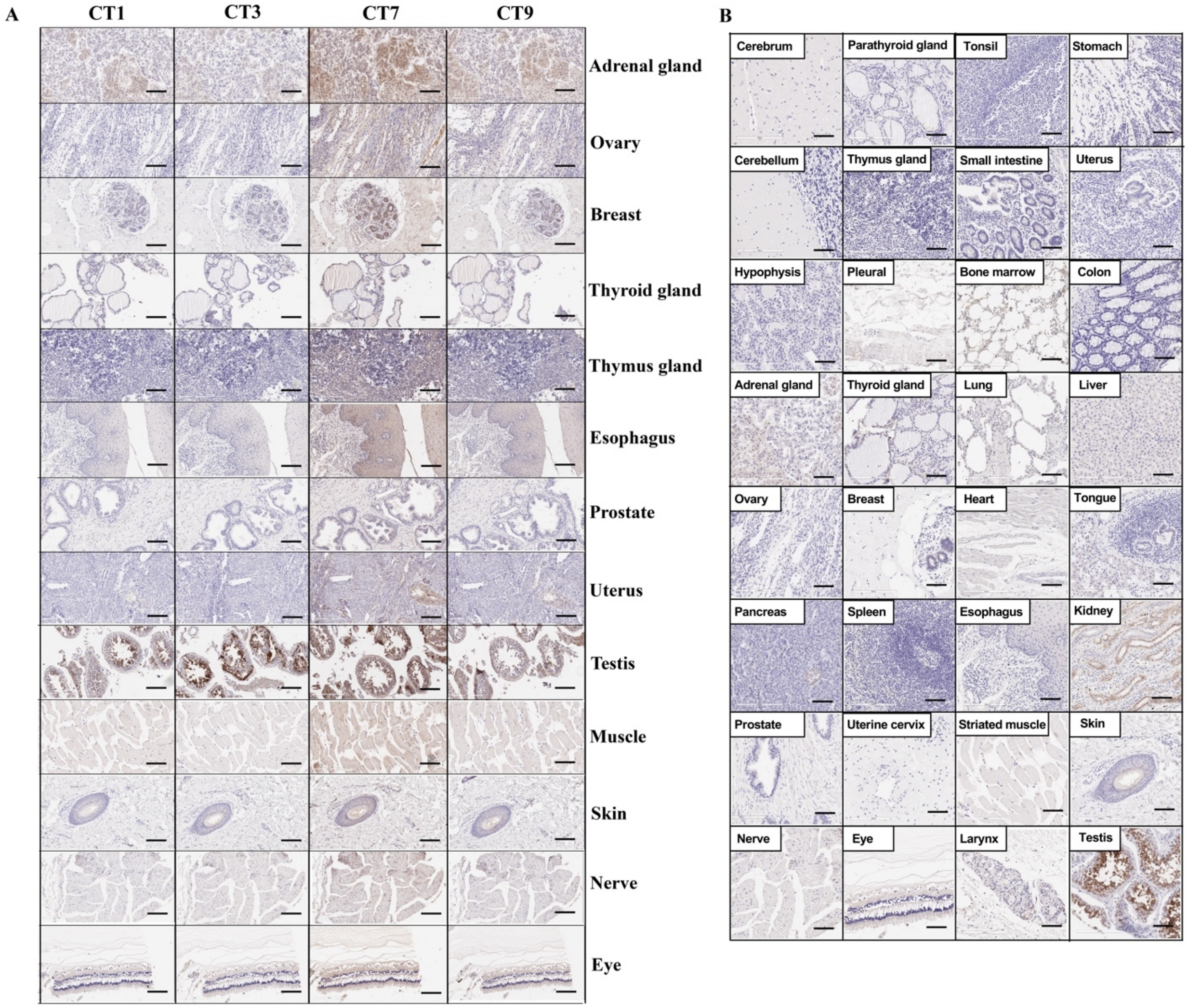


**Figure S1. GPC2 expression in normal human tissues as determined by immunohistochemistry. Related to Figure 1.** (A) The GPC2 immunostaining by several antibodies including CT1, CT3, CT7 and CT9. (B) The GPC2 immunostaining by CT3 antibody on 32 normal human tissues. The tissues were labeled with 1 μg/ml antibody. Scale bar = 200 μm. The information of each specimen can be found in Table S1.

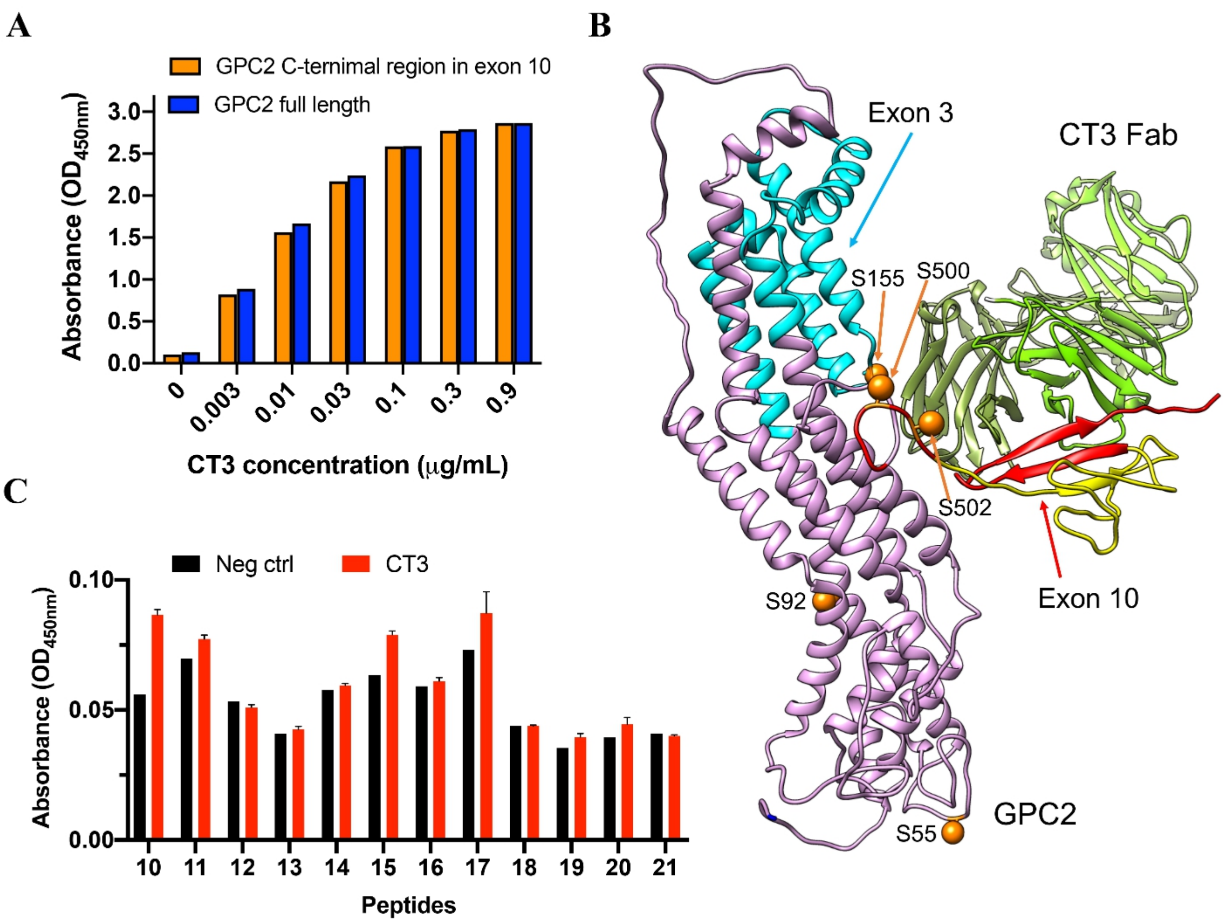


**Figure S2. CT3 binds to both exon 3 and exon 10 of GPC2. Related to Figure 3.** (A) CT3 reacts to the C-terminal region in exon 10 of GPC2 and full length GPC2 equally well. Various concentrations of CT3 were used for ELISA. (B) A ribbon diagram in which GPC2 and CT3 Fab are placed together. 5 predicted HS attachment sites are labelled at S55, S92, S155, S500 and S502. The CT3 light chain, heavy chain, GPC2 exon 3, and GPC2 C-terminal peptide in exon 10 are colored in light green, olive green, cyan and yellow, respectively. The full exon 10 is colored in both red and yellow. (C) CT3 shows no binding to single linear peptide in exon 3 of GPC2. 1 μg/ml of CT3 was used for ELISA.

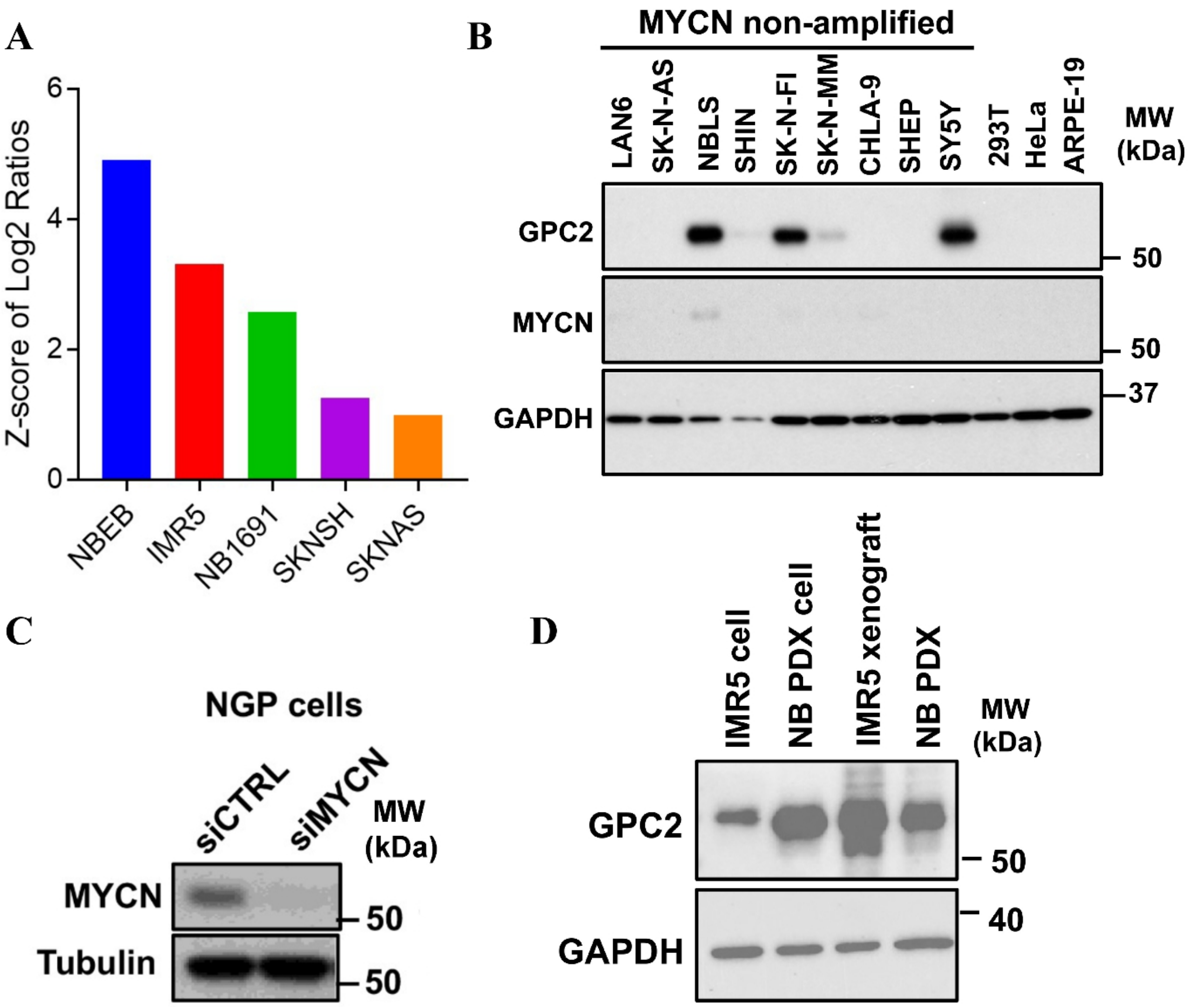


**Figure S3. GPC2 expression in neuroblastoma cell lines and PDX. Related to Figure 4.** (A) *GPC2* mRNA expression in neuroblastoma cell lines. (B) Western blotting with CT3 was performed to detect GPC2 protein expression in 9 *MYCN* non-amplified neuroblastoma cell lines (LAN6, SK-N-AS, NBLS, SHIN, SK-N-FI, SK-N-MM, CHLA-9, SHEP, SY5Y), as well as non-neuroblastoma cell lines (293T, HeLa, and ARPE-19). (C) The specificity of the anti-N-Myc antibody was demonstrated in knockdown of *MYCN* in NGP cells. (D) GPC2 protein expression is appreciably higher in neuroblastoma PDX and IMR5 xenograft than IMR5 cells cultured as monolayers *in vitro*. Neuroblastoma PDX: SJNBL012407

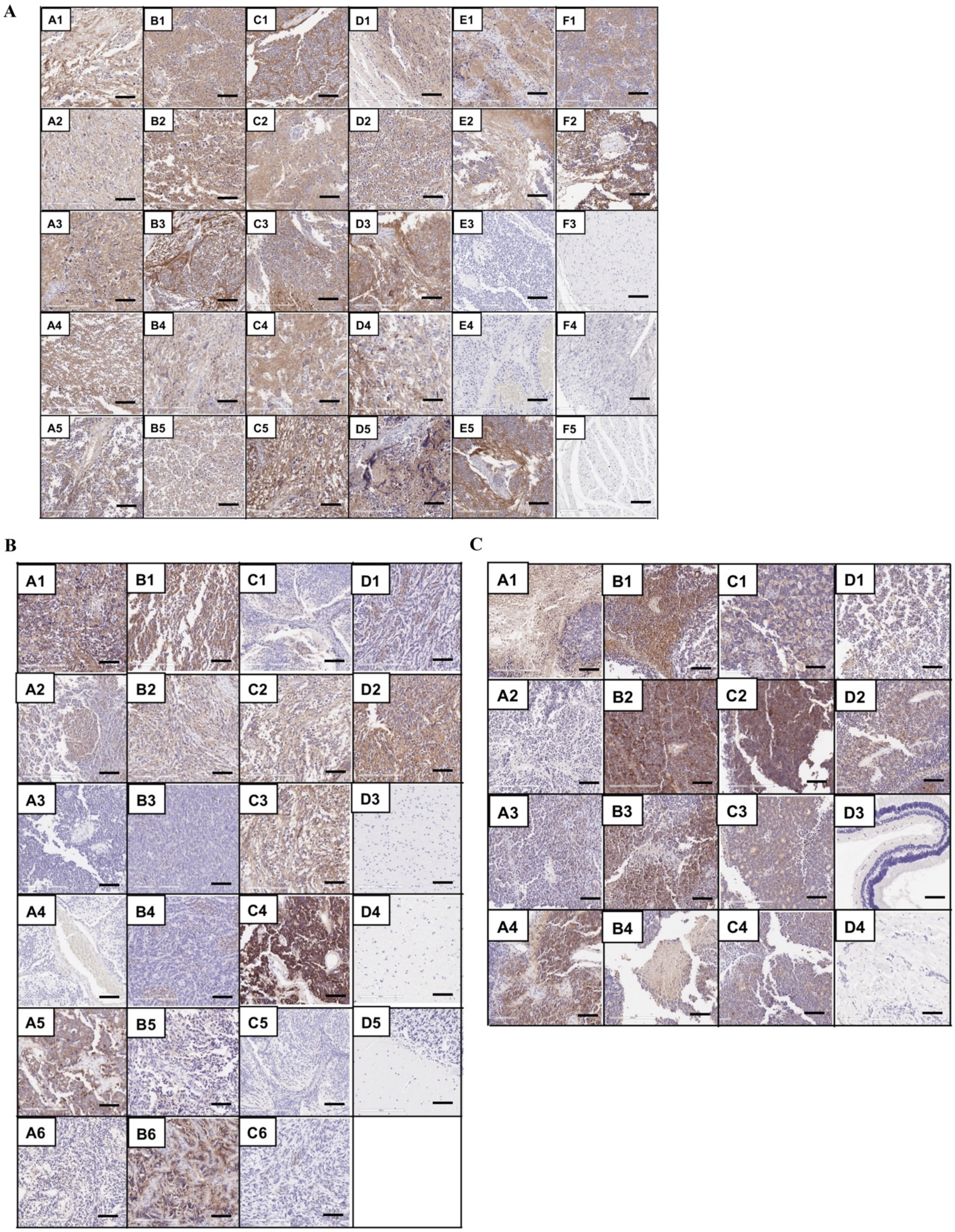


**Figure S4. GPC2 expression in pediatric cancers as determined by immunohistochemistry. Related to Figure 4.** (A) Strong GPC2 staining was found in 95% (19/20) cases of neuroblastoma tissues (A1 through D5). E1 through

E5 are normal nerve tissues. (B) Strong GPC2 staining was found in 64% (7/11) cases of medulloblastoma tissues (A1 to C1). C2 to C4 are normal brain tissues. (C) Strong GPC2 staining was found in 78% (7/9) cases of retinoblastoma tissues (A1 to C1). C2 to C4 are cancer adjacent normal eye tissues. All tissues were labeled with 1 μg/ml CT3. Scale bar = 200 μm. The information of each specimen can be found in Table S4.

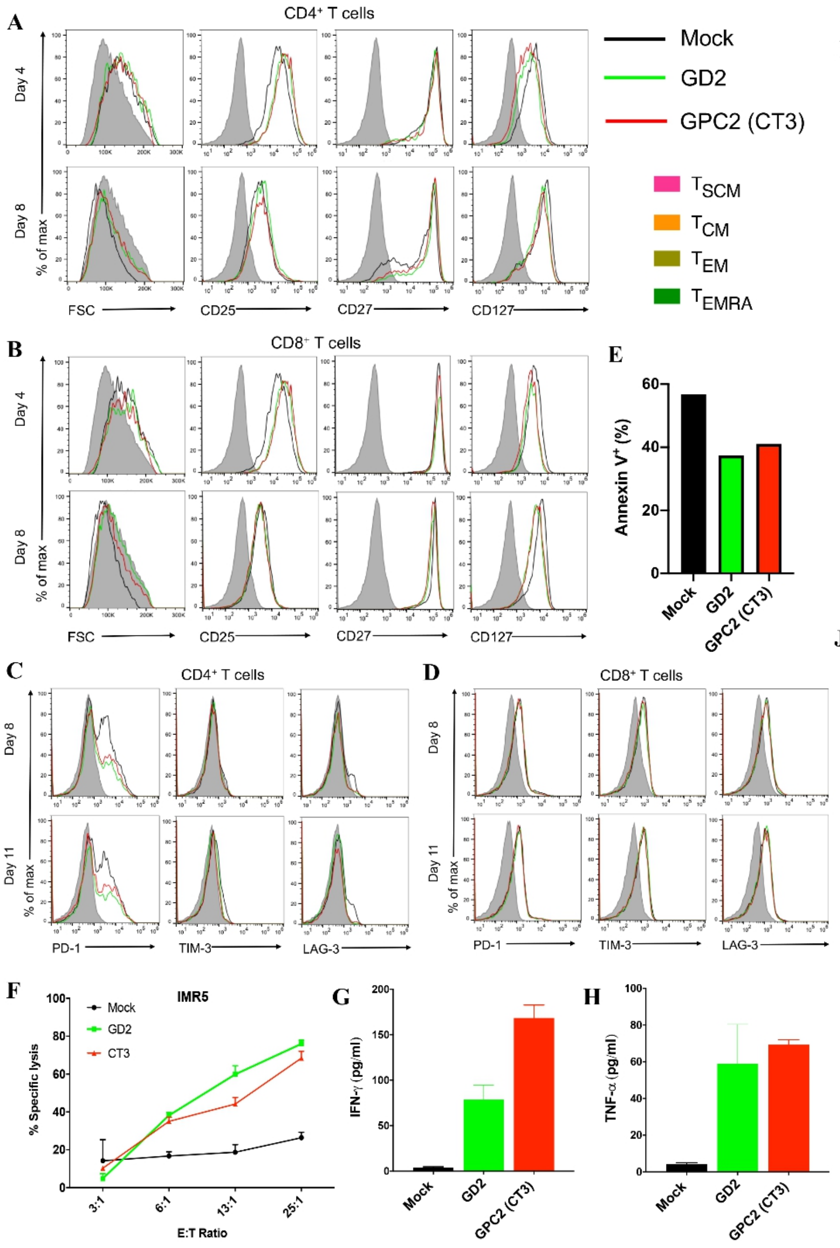

**Figure S5. CT3 CAR T cells exhibit low level of tonic signaling during *ex vivo* expansion. Related to Figure 5.** (A-B) activation marker expression after initial activation in CD4$^{+}$ (A) and CD8$^{+}$ (B) T cell populations. (C-D) exhaustion marker expression after initial activation in CD4$^{+}$ (C) and CD8$^{+}$ (D) T cell populations. (E) Quantification of apoptosis of CAR T cells 11 days after initial activation. (F) cytolytic activity of GD2 and GPC2 (CT3) CAR T cells in IMR5 cells after 24 hours of co-culture. (G-H) production of IFN-γ (G) and TNF-α (H) of CAR T cells co-incubated with IMR5 cells at the E:T ratio of 6:1. (I-J) Memory T cell subsets of unstimulated PBMCs, mock T cells, GD2 and GPC2 (CT3) CAR T cells. (I) relative proportion of stem cell-like memory ($T_{SCM}$),

central memory ($T_{CM}$), effector memory ($T_{EM}$), and terminally differentiated effector memory ($T_{EMRA}$) subsets defined by CD62L, CD45RA and CD95 expression in the $CD4^+$ T cell population. (J) relative proportion of $T_{SCM}$, $T_{CM}$, $T_{EM}$, and $T_{EMRA}$ subsets in the $CD8^+$ T cell population.

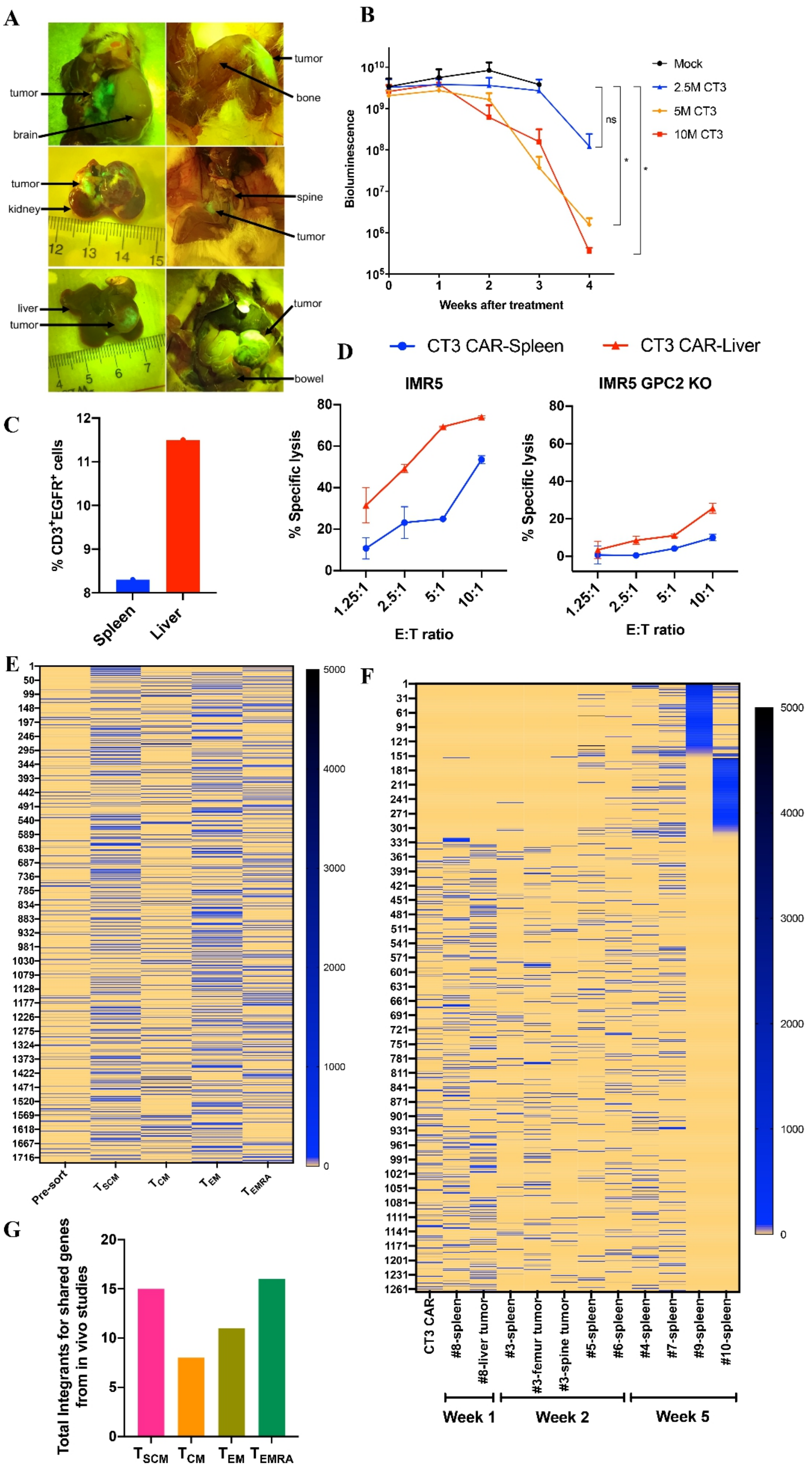
A
tumor
brain
tumor
bone
tumor
kidney
spine
tumor
liver
tumor
tumor
bowel
B
Bioluminescence
Weeks after treatment
Mock
2.5M CT3
5M CT3
10M CT3
ns
*
*
C
% CD3+EGFR+ cells
Spleen
Liver
D
CT3 CAR-Spleen
CT3 CAR-Liver
IMR5
IMR5 GPC2 KO
% Specific lysis
1.25:1
2.5:1
5:1
10:1
E:T ratio
E
1
50
99
148
197
246
295
344
393
442
491
540
589
638
687
736
785
834
883
932
981
1030
1079
1128
1177
1226
1275
1324
1373
1422
1471
1520
1569
1618
1667
1716
Pre-sort
TSCM
TCM
TEM
TEMRA
5000
4000
3000
2000
1000
0
F
1
31
61
91
121
151
181
211
241
271
301
331
361
391
421
451
481
511
541
571
601
631
661
691
721
751
781
811
841
871
901
931
961
991
1021
1051
1081
1111
1141
1171
1201
1231
1261
CT3 CAR
#8-spleen
#8-liver tumor
#3-spleen
#3-femur tumor
#3-spine tumor
#5-spleen
#6-spleen
#4-spleen
#7-spleen
#9-spleen
#10-spleen
Week 1
Week 2
Week 5
G
Total Integrants for shared genes from in vivo studies
TSCM
TCM
TEM
TEMRA

**Figure S6. IMR5 xenograft bearing mice treated with CT3 CAR T cells in Figure 6A-C, and distribution of CT3 CAR integration sites. Related to Figure 6.** (A) IMR5 tumor cells metastasized to various sites including brain, spine, femur, etc. (B) the averaged tumor bioluminescence of all groups including mock T cells, 2.5 million CT3 CAR T cells, 5 million CT3 CAR T cells and 10 million CT3 CAR T cells. (C) Frequencies of $CD3^+hEGFRt^+$ cells representing CT3 CAR T cells in spleen and liver from one treated mouse. (D) cytolytic activity of CT3 CAR T cells recovered from mouse spleen and liver against IMR5 cells as well as GPC2 KO-IMR5 cells after 24 hours of co-culture. ns: not significant; *P<0.05 (E) integration sites were randomly distributed among cultured CT3 CAR T cells. Memory T cells include central memory ($T_{CM}$), stem cell-like memory ($T_{SCM}$), terminally differentiated effector memory ($T_{EMRA}$), and effector memory ($T_{EM}$) subsets defined by CD62L, CD45RA and CD95 expression. (F) distribution of integration sites in spleen and IMR5 tumors of mice treated with CT3 CAR T cells at week 1, week 2 and week 5 post-infusion seen in Figures 6A-C. (G) The shared integration genes from *in vivo* studies were detected in different memory T cell subsets from *in vitro* culture as seen in Table S5. The times of every founded gene were added together in each subset.

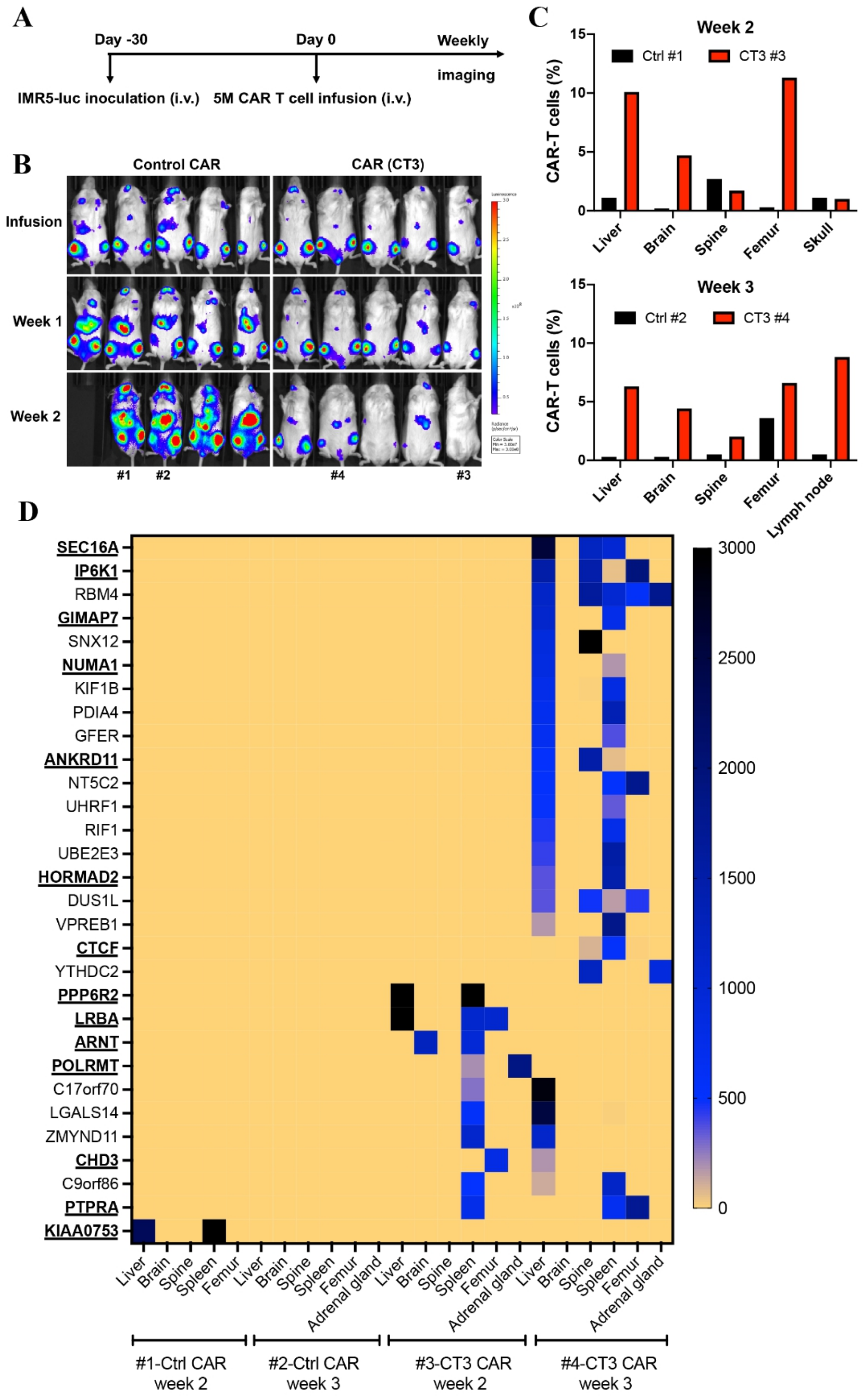


**Figure S7. CT3 CAR T cells regress tumor growth in the IMR5 xenograft mouse model. Related to Figure 6.**

(A) Schematic of the metastatic IMR5 xenograft mouse model. IMR5 tumor-bearing NSG mice were i.v. injected with 5 million control CAR T cells targeting glypican 3 and CT3 CAR T cells. (B) CT3 CAR T cells reduced tumor

burden in mice 2 weeks post-infusion. (C) CAR vector-positive cells were detected in various tissues from the same mouse receiving CT3 CAR T cells at week 2 and week 3 post-infusion. Minimal CAR vector-positive cells were found in mice infused with control CAR T cells targeting GPC3. (D) Distribution of integration sites in mice treated with control CAR T cells and CT3 CAR T cells. The integrated genes were largely shared in T cells recovered from various tissues of the same mouse, while some overlap was also observed in different mice receiving treatment. None of the shared integrated sites were found in mice from the control CAR group. The genes found in the memory T cell subsets shown in Table S5 are bolded and underlined.
